# Association between vitamin D receptor (VDR) polymorphisms and the risk of multiple sclerosis (MS): an updated meta-analysis

**DOI:** 10.1186/s12883-019-1577-y

**Published:** 2019-12-26

**Authors:** Danyal Imani, Bahman Razi, Morteza Motallebnezhad, Ramazan Rezaei

**Affiliations:** 1 Department of Immunology, School of Public Health, Tehran University of Medical Sciences(TUMS), Tehran, Iran; 2 Department of Hematology and Blood Banking, School of Medicine, Tarbiat modares university (TMU), Tehran, Iran; 3 Immunology Research Center, Tabriz University of Medical Sciences, Tabriz, Iran; 4 Student Research Committee, Tabriz University of Medical Sciences, Tabriz, Iran; 5 Department of Immunology, Faculty of Medicine, Iran University of Medical Sciences, Tehran, Iran; 6 Department of Immunology, School of Medicine, Shahid Beheshti University of Medical Sciences, Tehran, 14194 Iran

**Keywords:** Vitamin D receptor, Multiple sclerosis, Polymorphism, Meta-analysis

## Abstract

**Background:**

The association between the *Vitamin D Receptor* (*VDR*) gene polymorphism and the risk of Multiple sclerosis (MS) has been evaluated in several researches. However, the findings were inconsistent and inconclusive. Therefore, we set out a meta-analysis of all eligible published case-control studies to obtain an exact evaluation of the association between *VDR* gene polymorphisms and MS.

**Method:**

All relevant studies reporting the association between the *VDR* gene FokI (rs2228570), or/and TaqI (rs731236) or/and BsmI (rs1544410) or/and ApaI (rs7975232) polymorphisms and susceptibility to MS published up to May, 2019 were identified by comprehensive systematic search in the electronic database of web of science, Scopus, and PubMed. After that, the strength of association between *VDR* gene polymorphisms and susceptibility to MS was evaluated by odds ratio (OR) and 95% confidence interval (CI).

**Results:**

A total of 30 case–control studies were included in the meta-analysis. The overall results suggested a significant association between TaqI polymorphism and MS risk under heterozygote genetic model (OR = 1.27, 95%CI = 1.01–1.59, random effect). Moreover, the pooled results of subgroup analysis declined presence of significant association under all defined genetic model. In subgroup analysis, BsmI polymorphisms was associated with increased risk of MS under recessive model in Asian populations. On the other hand, ApaI polymorphism was associated with decreased risk of MS under recessive and aa vs. AA model in Asian populations.

**Conclusion:**

This meta-analysis suggested a significant association between TaqI polymorphism and MS susceptibility. Furthermore, BsmI polymorphism was associated with increased risk of MS in Asian populations. In contrast, ApaI polymorphism was associated with decreased risk of MS in Asian populations. Future large-scale studies on gene–environment and gene–gene interactions are required to estimate risk factors and assist early diagnosis of patients at high risk for MS.

## Background

Multiple sclerosis (MS) is a chronic and demyelinating disorder of the brain and spinal cord that mainly develops in young individuals [1, 2]. Tissue damage in MS results from a dynamic and complex interaction between the glia (oligodendrocytes and their precursors, astrocytes, and microglia), neurons, and immune system. Although there is an argument about whether the original cause of MS is extrinsic or intrinsic to the CNS, several researches in animal models in combination with evaluation of immune cells in humans have elucidated a fundamental function for adaptive immunity [3, 4]. It has been demonstrated that genetic and environmental factors play important roles in susceptibility to the disease [5]. Vitamin D is a group of fat-soluble secosteroids that have functional and regulatory effects in the body. Vitamin D has been implicated in the development of the brain and spinal cord. Alternatively, the active form of vitamin D, 1,25-dihydroxyvitamin D has a wide anti-inflammatory and immunomodulatory properties [6, 7]. Vitamin D exerts its immunomodulatory functions within the immune system by decreasing the presentation of major histocompatibility complex (MHC) II on T cells and monocytes. Vitamin D also reduces T cell proliferation and pro-inflammatory cytokine release [8]. The lower serum vitamin D levels compared to healthy controls have been reported in MS patients. Moreover, Vitamin D has positive effects in regulating MS risk development [9, 10]. The effects of Vitamin D on the immune system are exerted by binding to the nuclear Vitamin D Receptor (VDR) [11]. Particular variants of the VDR gene are related to changes in vitamin D metabolism and function [12]. Taken together, these results suggested that VDR may play an important role in the pathogenesis of MS.

The human *VDR* gene is located on the chromosome 12q12–14 and series of restriction fragment length polymorphisms (RFLP) in the human *VDR* gene have been reported, containing BsmI (rs1544410), ApaI (rs7975232), FokI (rs2228570), and TaqI (rs731236) restriction sites [13]. ApaI, BsmI, and TaqI are localized near the 3′-untranslated region (UTR) of the *VDR* gene in the intron between exons 8 and 9, and shown to be in strong linkage disequilibrium (LD) with each other [14]. The 3′-UTR of the *VDR* gene is involved in the regulation of gene expression by regulating the mRNA stability and expression level [15]. Polymorphism FokI is located at the translation starting codon [16].

The association between MS and *VDR* gene single nucleotide polymorphisms (SNPs) has been investigated in several studies. Particularly, studies have evaluated associations between the most common SNPs of the *VDR* gene (TaqI, ApaI, FokI, and BsmI polymorphisms) and MS. While studies in Australia [17], Kuwait [18], and southeast of Iran [19] reported a significant association between TaqI, ApaI, and FokI polymorphisms and MS, other studies in Tunisia [20], Slovakia [21], and Greece [22] have failed to find such association. The reasons for this disparity may be small sample sizes, low statistical power, clinical heterogeneity, or a combination of these factors. To offset these limitations, this meta-analysis was performed to investigate whether VDR gene polymorphisms contribute to MS or not. Up to now, there are four meta-analysis which investigated the association between VDR polymorphisms and MS. The two studies performed by Huang et al. [23]., and Garcia-Martin et al. [24]. have indicated that there was no association between *VDR* gene polymorphism and MS risk. Nevertheless, the other meta-analysis by Zhang et al. [25]., and Tizaoui et al. [26]. demonstrated a significant association between ApaI and FokI and MS susceptibility. Since publishing of the last meta-analysis, seven new studies have been founded in electronic databases. Therefore, we conducted a meta-analysis of all eligible published case-control studies to obtain an exact evaluation of the association between *VDR* gene polymorphisms and susceptibility to MS.

## Methods

The current systematic review and meta-analysis was conducted according to the Preferred Reporting Items for Systematic Reviews and Meta-Analyses (PRISMA) statement [27].

### Publication search

All relevant studies reporting the association between the *VDR* gene FokI (rs2228570) or/and TaqI (rs731236) or/and BsmI (rs1544410) or/and ApaI (rs7975232) polymorphisms and susceptibility to MS published up to May 2019 were identified by comprehensive systematic search in the electronic database of web of science, Scopus, and PubMed. The following search terms were applied: (VDR” or “vitamin D receptor”) AND (“multiple sclerosis” OR “MS”) AND (“polymorphisms” OR “single nucleotide” OR “polymorphism” OR “SNP” OR “variation” OR “mutation”). As a complementary approach, in order to detect additional potentially relevant studies, manual evaluation of the reference list of the included eligible studies was performed. In this meta-analysis, the strategy of search was restricted solely to the English-language publications and human population.

### Study selection

Two reviewers independently assessed titles and abstract of all studies retrieved in the initial search. Articles not following the eligibility criteria were excluded by applying a hierarchical approach based on study design. Full-text examination was applied if we could not decide include or exclude based on titles and abstract. In particular conditions, if an author has published more than one study by the same case series, the most recently published study was included. Any disagreements were discussed and resolved by consensus.

### Eligibility criteria

Studies considered eligible if meet the following criteria: 1) All eligible case–control studies that evaluate the relationship between the *VDR* gene single nucleotide polymorphisms and the risk of MS as the main outcome; 2) Sufficient data are available to extract or calculate odds ratios (ORs) and 95% confidence intervals (CI); 3) Contained genotypic or allelic distributions of case and healthy individuals for *VDR* gene polymorphism in the studies. The exclusion criteria were as follows: 1) Studies which genotype or allelic frequency could not be extracted; 2) Letters, case reports, reviews, comments, book chapter, and abstracts; 3) Duplicated reports and studies with repetitive subjects. The application of these criteria yielded 30 case–control studies eligible for the meta-analysis.

### Data extraction

Two reviewers independently extracted all data according to standardized extraction form for the following data: The author’s name, journal and year of publication, country of origin, ethnicity, number of cases and controls for every gender separately, mean or range of age, genotyping method, total sample size of cases and controls, and the number of cases and controls for each genotype. For quality assessment of the included publications, the Newcastle-Ottawa Scale (NOS) was applied [28]. Studies with scores 0–3, 4–6 or 7–9 were of low, moderate or high-quality, respectively.

### Statistical analysis

Deviation from Hardy–Weinberg equilibrium (HWE) for distribution of the allele frequencies was analyzed using Chi-Square test in control group. Sensitivity analysis was conducted to estimate the stability of the results by removing the studies not in HWE. The strength of association between the *VDR* gene FokI, TaqI, BsmI, and ApaI polymorphisms and susceptibility to MS was evaluated by OR and 95% CI. Defined model for FokI, TaqI, BsmI, ApaI were as follow, respectively: **FokI**; dominant model (ff + Ff vs. FF), recessive model (ff vs. Ff + FF), allelic model (f vs. F), homozygote model (ff vs. FF), and heterozygote model (Ff vs. FF); **TaqI**; dominant model (tt + Tt vs. TT), recessive model (tt vs. Tt + TT), allelic model (t vs. T), homozygote model (tt vs. TT), and heterozygote model (Tt vs. TT); **BsmI**; dominant model (bb + Bb vs. BB), recessive model (bb vs. Bb + BB), allelic model (b vs. B), homozygote model (bb vs. BB), and heterozygote model (Bb vs. BB); **ApaI**; dominant model (aa+Aa vs. AA), recessive model (aa vs. Aa+AA), allelic model (a vs. A), homozygote model (aa vs. AA), and heterozygote model (Aa vs. AA). For each genetic model, subgroup analysis was applied to evaluate ethnicity effects. In consideration of the possibility of heterogeneity (between study variability) across included studies, chi-square based Q-test was used [29]. Additionally, to show possible heterogeneity quantitatively, the other index (*I*^*2*^) was calculated as the percentage of heterogeneity. There was significant heterogeneity if an *I*^*2*^ values exceeded 50% or the Q statistic had a *P* value less than 0.1. In the presence of significant heterogeneity, the random effects model (DerSimonian–Laird approach) was performed. Otherwise, the fixed effects model (Mantel–Haenszel approach) was performed for combination of data [30, 31]. Visual inspection of asymmetry in funnel plots asymmetry, Begg’s test, and Egger’s test were conducted to evaluate publication bias (*P* value< 0.05 was considered statistically significant) [32, 33]. The data analyses were carried out using STATA (version 14.0; Stata Corporation, College Station, TX) and SPSS (version 23.0; SPSS, Inc. Chicago, IL).

## Results

### Study characteristics

**Fig. 1:**
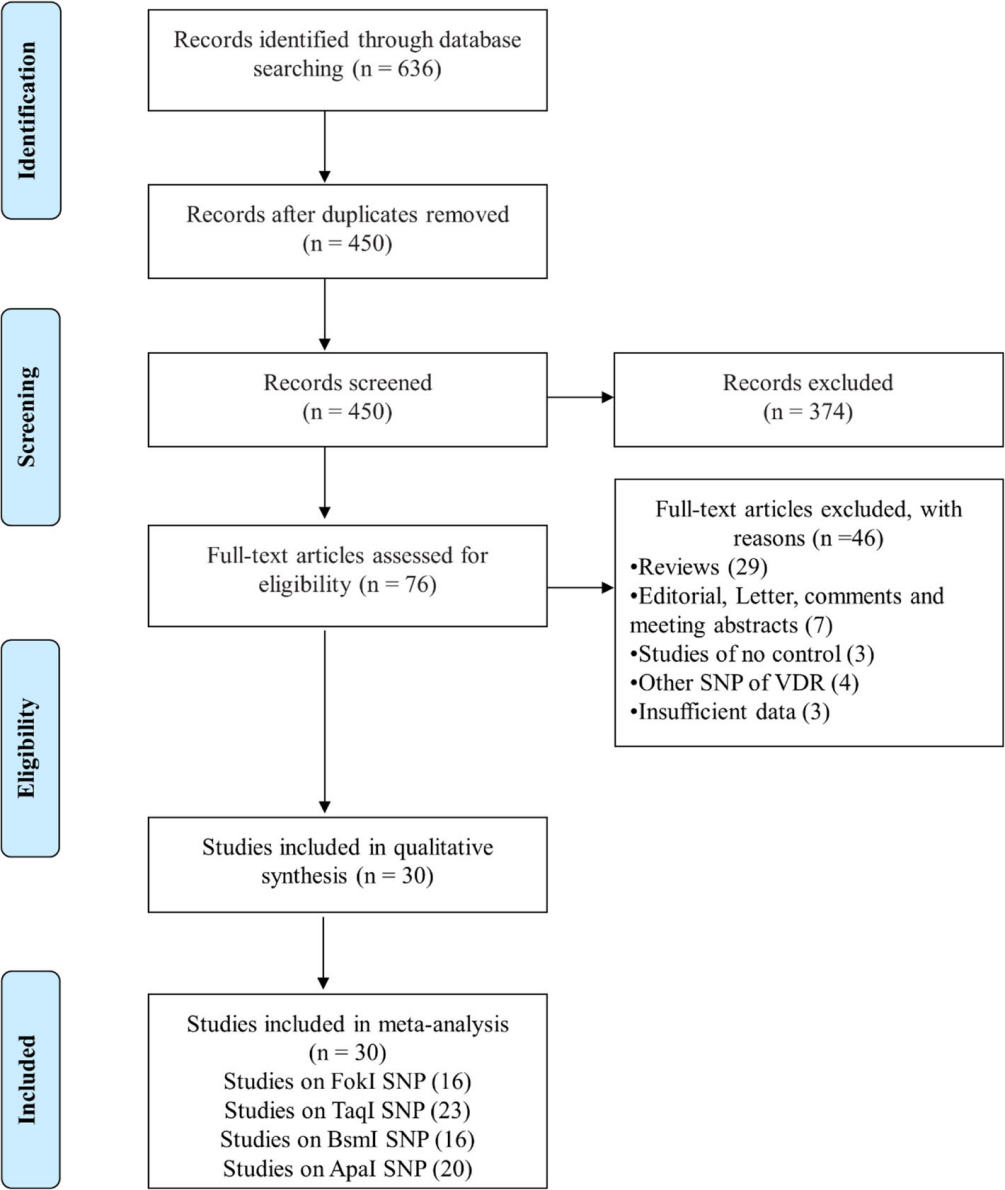
Flow diagram of study selection process

The primary search in web of science, Scopus, and PubMed databases yielded a total of 636 publications. After removal of duplicates and evaluation of title/abstract, only 76 studies remained for full-text examination. Eventually, 30 studies met the inclusion criteria and included for quantitative synthesis. The search workflow is shown in Fig. 1. Study characteristics are summarized in Table 1. Among 30 eligible studies, 16 Studies investigated FokI SNP, 23 Studies TaqI SNP, 16 studies BsmI SNP and 20 Studies ApaI SNP. The studies were published between 1999 and 2019. Taq-Man and PCR-RFLP genotyping methods were used by the most studies.

**Table 1:** Characteristics of studies included in Meta-analysis of overall MS

Study author	Year	Country	Ethnicity	Sex cases/controls	Total cases/control	Age case/control (Mean)	Genotyping method	Quality score
FokI (rs2228570)								
Partridge et al.	2004	UK	European	M = NR F=NR	406 /234	43.8 ± 11.2 / 50.1	NR	7
Tajouri et al.	2005	Australia	Australian	M = NR F=NR	98 / 93	NR	PCR–RFLP	6
Smolders et al.	2009	Netherland	European	M = 62/ 142 F = 150/ 147	212 / 289	46.7 ± 11.9 /34.9 ± 14.3	PCR–RFLP	8
Dickinson et al.	2009	Australia	Australian	M = NR F=NR	136 / 235	43.5 ± 9.3/ 43.6 ± 9.2	Taq-Man	7
Simon et al.	2010	USA	American	M = NR F=NR	100 / 100	NR	Taq-Man	7
Garcia-Martin et al.	2013	Spain	European	M = 94/ 98 F = 209/ 212	303 / 310	43.9 ± 11.4/ 43.4 ± 11.7	Taq-Man	8
Al-Temaimi et al.	2015	Kuwait	Asian	M = 17/ 19 F = 33/ 31	50 / 50	33.4 ± 9.6/ 28.6 ± 7.9	Taq-Man	7
Narooie-Nejad et al.	2015	Iran	Asian	M = 25 / 28 F = 88/ 94	113 / 122	32.4 ± 8.9/ 30.8 ± 10.2	PCR–RFLP	7
Cierny et al.	2015	Slovakia	European	M = 66 / 74 F = 204 / 229	270 / 303	41.3 ± 10.8/ 38.7 ± 13.6	PCR–RFLP	8
Luisa Agnello et al.	2016	Italy	European	M = 24 / 30 F = 80 / 42	104 / 75	39.6 ± 10.3/ 45.2 ± 9.36	PCR–RFLP	6
Abdollahzadeh et al.	2016	Iran	Asian	M = 40 / 38 F = 120 / 112	160 / 150	35.9 ± 3 / 36.8 ± 1.8	PCR–RFLP	7
Yucel et al.	2017	Turkey	European	M = NRF=NR	29 / 114	33.7 ± 10.7 / 33.1 ± 8.5	Taq-Man	6
Bettencourt et al.	2017	Portugal	European	M = 185/198 F = 348/248	533 / 446	30.2 ± 9.3/ NR	Taq-Man	8
Kamisli et al.	2018	Turkey	European	M = 46 / 58 F = 121 / 88	167 / 146	39.96 ± 9.4 / 33.81 ± 7.1	Taq-Man	7
Sadeghi et al.	2018	Iran	Asian	M = 17/11 F = 63/39	80 / 50	18–60/ 18–60	PCR–RFLP	6
Křenek et al.	2018	Czech Republic	European	M = 80/49 F = 216/86	296/135	34–37 / NR	PCR–RFLP	7
TaqI (rs731236)								
Partridge et al.	2004	UK	European	M = NR F=NR	402 /231	43.8 ± 11.2 / 50.1	NR	7
Tajouri et al.	2005	Australia	Australian	M = NR F=NR	104 / 186	NR	PCR–RFLP	6
Smolders et al.	2009	Netherland	European	M = 62/ 142 F = 150/ 147	212 / 289	46.7 ± 11.9 /34.9 ± 14.3	PCR–RFLP	8
Dickinson et al.	2009	Australia	Australian	M = NR F=NR	136 / 235	43.5 ± 9.3/ 43.6 ± 9.2	Taq-Man	7
Simon et al.	2010	USA	American	M = NR F=NR	100 / 100	NR	Taq-Man	7
Sioka et al.	2011	Greece	European	M = 23/ 23 F = 46/ 58	69 / 81	39 ± 10.5/38.7 ± 10.7	Taq-Man	7
Agliardi et al.	2011	Italy	European	M = NR F=NR	641 / 558	27.8 ± 9.2/ 29.4 ± 6.5	Taq-Man	8
Irizar et al.	2012	Spain	European	M = NR F=NR	136 / 337	44.14 ± 13.02/ 50.17 ± 13.26	PCR-SSP	7
Garcia-Martin et al.	2013	Spain	European	M = 94/ 98 F = 209/ 212	303 / 310	43.9 ± 11.4/ 43.4 ± 11.7	Taq-Man	8
Selma et al.	2015	Tunisia	African	M = 22/ 47 F = 38/ 67	60 / 114	35.8 ± 6.3 / 37 ± 9.3	PCR–RFLP	6
Narooie-Nejad et al.	2015	Iran	Asian	M = 25 / 28 F = 88/ 94	113 / 122	32.4 ± 8.9/ 30.8 ± 10.2	PCR–RFLP	7
Al-Temaimi et al.	2015	Kuwait	Asian	M = 17/ 19 F = 33/ 31	50 / 50	33.4 ± 9.6/ 28.6 ± 7.9	Taq-Man	7
Yamout et al.	2016	Lebanon	Asian	M = NR F=NR	50 / 99	32.3 ± 12.7 / 35.2 ± 13	PCR–RFLP	6
Cierny et al.	2016	Slovakia	European	M = 66 / 74 F = 204 / 229	270 / 303	41.3 ± 10.8/ 38.7 ± 13.6	PCR–RFLP	8
Luisa Agnello et al.	2016	Italy	European	M = 24 / 30 F = 80 / 42	104 / 75	39.6 ± 10.3/ 45.2 ± 9.36	PCR–RFLP	6
Terzi et al.	2016	Turkey	European	M = NR F=NR	87 / 99	30.14 9.66/ NR	PCR–RFLP	6
Abdollahzadeh et al.	2016	Iran	Asian	M = 40 / 38 F = 120 / 112	160 / 150	35.9 ± 3 / 36.8 ± 1.8	PCR–RFLP	7
Yucel et al.	2017	Turkey	European	M = NR F=NR	28 / 72	33.7 ± 10.7 / 33.1 ± 8.5	Taq-Man	6
Kamisli et al.	2018	Turkey	European	M = 46 / 58 F = 121 / 88	167 / 146	39.96 ± 9.4 / 33.81 ± 7.1	Taq-Man	7
Morales et al.	2018	Mexico	American	M = 39/ 57 F = 81/123	120 / 180	33.89 ± 10.03/ 32.79 ± 10.17	Taq-Man	6
Sadeghi et al.	2018	Iran	Asian	M = 17/11 F = 63/39	80 / 50	18–60/ 18–60	PCR–RFLP	6
Cakina et al..	2018	Turkey	European	M = 19/22 F = 51/48	70 / 70	44.4 ± 11.2 / 38.2 ± 9.5	PCR–RFLP	7
Křenek et al.	2018	Czech Republic	European	M = 80/49 F = 216/86	296 / 135	34–37 / NR	PCR–RFLP	7
BsmI (rs1544410)								
Fukazawa et al.	1999	Japan	Asian	M = 21/33 F = 56/62	77 / 95	34.9 ± 12.9/NR	PCR–RFLP	8
Qinli Sun et al.	2004	China	Asian	M = NR F=NR	63 / 95	NR	PCR–RFLP	6
Bing Wu et al.	2009	China	Asian	M = NR F=NR	83 / 120	NR	PCR–RFLP	7
Simon et al.	2010	USA	American	M = NR F=NR	101 / 100	NR	Taq-Man	7
Sioka et al.	2011	Greece	European	M = 23/ 23 F = 46/ 58	69 / 81	39 ± 10.5/38.7 ± 10.7	Taq-Man	7
Al-Temaimi et al.	2015	Kuwait	Asian	M = 17/ 19 F = 33/ 31	50 / 50	33.4 ± 9.6/ 28.6 ± 7.9	Taq-Man	7
Narooie-Nejad et al.	2015	Iran	Asian	M = 25 / 28 F = 88/ 94	113 / 122	32.4 ± 8.9/ 30.8 ± 10.2	PCR–RFLP	7
Abdollahzadeh et al.	2016	Iran	Asian	M = 40 / 38 F = 120 / 112	160 / 150	35.9 ± 3 / 36.8 ± 1.8	PCR–RFLP	7
Yamout et al.	2016	Lebanon	Asian	M = NR F=NR	50 / 99	32.3 ± 12.7 / 35.2 ± 13	PCR–RFLP	6
Cierny et al.	2016	Slovakia	European	M = 66 / 74 F = 204 / 229	270 / 303	41.3 ± 10.8/ 38.7 ± 13.6	PCR–RFLP	8
Luisa Agnello et al.	2016	Italy	European	M = 24 / 30 F = 80 / 42	104 / 75	39.6 ± 10.3/ 45.2 ± 9.36	PCR–RFLP	6
Terzi et al.	2016	Turkey	European	M = NR F=NR	87 / 100	30.14 ± 9.66/ NR	PCR–RFLP	6
Morales et al.	2017	Mexico	American	M = 39/ 57 F = 81/123	120 / 180	33.89 ± 10.03/ 32.79 ± 10.17	Taq-Man	6
Sadeghi et al.	2018	Iran	Asian	M = 17/11 F = 63/39	80 / 50	18–60/ 18–60	PCR–RFLP	6
Cakina et al..	2018	Turkey	European	M = 19/22 F = 51/48	70 / 70	44.4 ± 11.2 / 38.2 ± 9.5	PCR–RFLP	7
Křenek et al.	2018	Czech Republic	European	M = 80/49 F = 216/86	296 / 135	34–37 / NR	PCR–RFLP	7
ApaI (rs7975232)								
Niino et al.	2000	Japan	Asian	M = 21 / 33 F = 56 / 62	77 / 95	36.2 ± 11.2 / 34.4 ± 10.2	PCR–RFLP	7
Qinli Sun et al.	2004	China	Asian	M = NR F=NR	63 / 95	NR	PCR–RFLP	6
Tajouri et al.	2005	Australia	Australian	M = NR F=NR	104 / 100	NR	PCR–RFLP	6
Smolders et al.	2009	Netherland	European	M = 62/ 142 F = 150/ 147	212 / 289	46.7 ± 11.9 /34.9 ± 14.3	PCR–RFLP	8
Bing Wu et al.	2009	China	Asian	M = NR F=NR	83 / 120	NR	PCR–RFLP	7
Simon et al.	2010	USA	American	M = NR F=NR	100 / 100	NR	Taq-Man	7
Irizar et al.	2012	Spain	European	M = NR F=NR	134 / 340	44.14 ± 13.02/ 50.17 ± 13.26	PCR-SSP	7
Narooie-Nejad et al.	2015	Iran	Asian	M = 25 / 28 F = 88/ 94	113 / 122	32.4 ± 8.9/ 30.8 ± 10.2	PCR–RFLP	7
Al-Temaimi et al.	2015	Kuwait	Asian	M = 17/ 19 F = 33/ 31	50 / 50	33.4 ± 9.6/ 28.6 ± 7.9	Taq-Man	7
Selma et al.	2015	Tunisia	African	M = 22/ 47 F = 38/ 67	60 / 114	35.8 ± 6.3 / 37 ± 9.3	PCR–RFLP	6
Yamout et al.	2016	Lebanon	Asian	M = NR F=NR	50 / 134	32.3 ± 12.7 / 35.2 ± 13	PCR–RFLP	6
Luisa Agnello et al.	2016	Italy	European	M = 24 / 30 F = 80 / 42	104 / 75	39.6 ± 10.3/ 45.2 ± 9.36	PCR–RFLP	6
Abdollahzadeh et al.	2016	Iran	Asian	M = 40 / 38 F = 120 / 112	160 / 150	35.9 ± 3 / 36.8 ± 1.8	PCR–RFLP	7
Cierny et al.	2016	Slovakia	European	M = 66 / 74 F = 204 / 229	270 / 303	41.3 ± 10.8/ 38.7 ± 13.6	PCR–RFLP	8
Terzi et al.	2016	Turkey	European	M = NR F=NR	87 / 100	30.14 9.66/ NR	PCR–RFLP	6
Yucel et al.	2017	Turkey	European	M = NR F=NR	26 / 81	33.7 ± 10.7 / 33.1 ± 8.5	Taq-Man	6
Kamisli et al.	2018	Turkey	European	M = 46 / 58 F = 121 / 88	167 / 146	39.96 ± 9.4 / 33.81 ± 7.1	Taq-Man	8
Sadeghi et al.	2018	Iran	Asian	M = 17/11 F = 63/39	80 / 50	18–60/ 18–60	PCR–RFLP	7
Cakina et al..	2018	Turkey	European	M = 19/22 F = 51/48	70 / 70	44.4 ± 11.2 / 38.2 ± 9.5	PCR–RFLP	7
Křenek et al.	2018	Czech Republic	European	M = 80/49 F = 216/86	296 / 135	34–37 / NR	PCR–RFLP	7

*NR* not reported, *M* male, *F* female, *MS* Multiple Sclerosis

### Quantitative synthesis

The distributions of FokI, TaqI, BsmI and ApaI genotypes of the included studies are shown in Table 2. FF for FokI SNP, TT for TaqI SNP, BB for BsmI SNP and AA for ApaI were used as the reference category. The heterogeneities in the comparisons (*I*^*2*^ < 50%, fixed-effects models; *I*^*2*^ > 50%, random-effects models) ascertained the application of Fixed-effects or random-effects models.

**Table 2:** Distribution of genotype and allele among MS patients and controls

Study author	MS cases					Healthy control					P-HWE	MAF
	FF	Ff	ff	F	f	FF	Ff	Ff	F	f		
FokI (rs2228570)												
Partridge et al.	155	196	55	506	306	83	105	46	271	197	0/22	0/42
Tajouri et al.	47	40	11	134	62	34	48	11	116	70	0/33	0/376
Smolders et al.	79	103	30	261	163	113	134	42	360	218	0/82	0/377
Dickinson et al.	58	61	17	177	95	86	110	39	282	188	0/72	0/4
Simon et al.	36	45	19	117	83	41	44	15	126	74	0/57	0/37
Garcia-Martin et al.	130	141	32	401	205	144	124	42	412	208	0/07	0/335
Al-Temaimi et al.	33	14	3	80	20	33	16	1	82	18	0/55	0/18
Narooie-Nejad et al.	73	32	8	178	48	93	29	0	215	29	0/13	0/118
Cierny et al.	96	143	31	335	205	118	143	42	379	227	0/89	0/374
Luisa Agnello et al.	34	52	18	120	88	29	36	10	94	56	0/82	0/373
Abdollahzadeh et al.	14	67	79	95	225	11	59	80	81	219	0/97	0/73
Yucel et al.	22	6	1	50	8	72	34	8	178	50	0/16	0/219
Bettencourt et al.	223	227	83	673	393	204	197	45	605	287	0/79	0/321
Kamisli et al.	75	77	15	227	107	94	46	6	234	58	0/92	0/198
Sadeghi et al.	47	32	1	126	34	20	26	4	66	34	0/26	0/34
Křenek et al.	102	145	49	349	243	37	74	24	148	122	0/21	0/451
Study author	MS cases					Healthy control					P-HWE	MAF
	TT	Tt	tt	T	t	TT	Tt	tt	T	T		
TaqI (rs731236)												
Partridge et al.	140	203	59	483	321	86	106	39	278	184	0/51	0/398
Tajouri et al.	27	57	20	111	97	104	42	40	250	122	0/57	0/327
Smolders et al.	83	96	33	262	162	112	138	39	362	216	0/53	0/373
Dickinson et al.	52	68	16	172	100	86	108	41	280	190	0/48	0/4
Simon et al.	40	50	10	130	70	36	48	16	120	80	1	0/4
Sioka et al.	30	30	9	90	48	33	36	12	102	60	0/67	0/37
Agliardi et al.	219	308	114	746	536	220	249	89	689	427	0/19	0/375
Irizar et al.	55	70	11	180	92	145	157	35	447	227	0/43	0/336
Garcia-Martin et al.	129	129	45	387	219	131	144	35	406	214	0/62	0/345
Selma et al.	28	29	3	85	35	75	38	1	188	40	0/1	0/175
Narooie-Nejad et al.	9	44	60	62	164	94	26	2	214	30	0/89	0/122
Al-Temaimi et al.	31	19	0	81	19	15	28	7	58	42	0/29	0/42
Yamout et al.	19	23	8	61	39	32	48	19	112	86	0/89	0/434
Cierny et al.	94	138	38	326	214	125	123	55	373	233	0/01	0/384
Luisa Agnello et al.	35	48	21	118	90	23	40	12	86	64	0/43	0/426
Terzi et al.	30	43	14	103	71	48	43	9	137	61	0/85	0/308
Abdollahzadeh et al.	38	80	42	156	164	63	68	19	194	106	0/92	0/353
Yucel et al.	13	15	0	41	15	31	26	15	88	56	0/05	0/388
Kamisli et al.	71	77	19	219	115	59	65	22	183	109	0/55	0/373
Morales et al.	65	46	9	176	64	122	41	17	285	75	0/005	0/208
Sadeghi et al.	38	41	1	117	43	14	34	2	62	38	0/02	0/38
Cakina et al.	20	41	9	81	59	20	45	5	85	55	0/001	0/392
Křenek et al.	118	151	27	387	205	58	66	11	182	88	0/19	0/325
Study author	MS cases					Healthy control					P-HWE	MAF
	BB	Bb	bb	B	b	BB	Bb	bb	B	B		
BsmI (rs1544410)												
Fukazawa et al.	0	11	66	11	143	3	24	68	30	160	0/62	0/842
Qinli Sun et al.	0	7	56	7	119	0	11	84	11	179	0/54	0/942
Bing Wu et al.	0	5	78	5	161	0	26	94	26	214	0/18	0/891
Simon et al.	39	49	13	127	75	34	47	19	115	85	0/71	0/425
Sioka et al.	28	41	0	97	41	26	55	0	107	55	0/004	0/339
Al-Temaimi et al.	20	30	0	70	30	15	25	10	55	45	0/94	0/45
Narooie-Nejad et al.	59	50	4	168	58	45	65	12	155	89	0/09	0/364
Abdollahzadeh et al.	46	79	35	171	149	70	65	15	205	95	0/98	0/316
Yamout et al.	10	21	19	41	59	16	53	30	85	113	0/35	0/57
Cierny et al.	43	139	88	225	315	73	111	119	257	349	0/001	0/575
Luisa Agnello et al.	23	48	33	94	114	17	37	21	71	79	0/92	0/526
Terzi et al.	19	40	28	78	96	14	47	39	75	125	0/97	0/625
Morales et al.	60	38	22	158	82	110	60	10	280	80	0/63	0/222
Sadeghi et al.	12	51	17	75	85	16	29	5	61	39	0/12	0/39
Cakina et al..	14	36	20	64	76	11	37	22	59	81	0/48	0/578
Křenek et al.	114	153	29	381	211	61	63	11	185	85	0/34	0/314
Study author	MS cases					Healthy control					P-HWE	MAF
	AA	Aa	aa	A	a	AA	Aa	aa	A	A		
ApaI (rs7975232)												
Niino et al.	21	23	33	65	89	9	41	45	59	131	0/93	0/689
Qinli Sun	9	17	37	35	91	15	29	51	59	131	0/005	0/689
Tajouri et al.	35	55	14	125	83	23	54	23	100	100	0/42	0/5
Smolders et al.	58	99	55	215	209	80	150	59	310	268	0/45	0/463
Bing Wu et al.	14	39	30	67	99	10	45	65	65	175	0/58	0/729
Simon et al.	29	45	26	103	97	28	50	22	106	94	0/97	0/47
Irizar et al.	39	60	35	138	130	76	178	86	330	350	0/37	0/514
Narooie-Nejad et al.	40	62	11	142	84	61	56	5	178	66	0/07	0/27
Al-Temaimi et al.	20	25	5	65	35	23	17	10	63	37	0/05	0/37
Selma et al.	14	36	10	64	56	40	58	16	138	90	0/48	0/394
Yamout et al.	19	22	9	60	40	33	51	15	117	81	0/51	0/503
Luisa Agnello et al.	31	58	15	120	88	26	41	8	93	57	0/16	0/38
Abdollahzadeh et al.	18	67	75	103	217	4	43	103	51	249	0/84	0/83
Cierny et al.	78	132	60	288	252	102	120	81	324	282	0/005	0/465
Terzi et al.	28	46	13	102	72	42	40	18	124	76	0/13	0/38
Yucel et al.	8	13	5	29	23	28	37	16	93	69	0/55	0/425
Kamisli et al.	62	76	29	200	134	54	67	25	175	117	0/59	0/4
Sadeghi et al.	22	53	5	97	63	23	22	5	68	32	0/93	0/32
Cakina et al..	28	27	15	83	57	20	21	29	61	79	0/001	0/564
Křenek et al.	27	183	86	237	355	31	78	26	140	130	0/07	0/481

*P-HWE p*-value for Hardy–Weinberg equilibrium, *MAF* minor allele frequency of control group

### Meta-analysis for FokI (rs2228570) polymorphism and MS

Overall 16 case-control studies with 3057 cases and 2852 controls were analyzed for assessment of FokI gene polymorphism and MS risk. Of 16 studies, 9 studies carried out in Europe continent [21, 24, 34–40] 4 studies in Asia continent [18, 19, 41, 42] one study in America continent [43] and finally 2 studies in Australia [17, 44] (Table 1). No significant association was observed between FokI polymorphism and MS risk across all genetic models. Additionally, subgroup analysis based on geographical location was performed which the pooled results rejected any association between FokI polymorphism and risk of MS in European and Asian populations. Since there was only one study for American, and two studies for Australian populations, these studies were excluded from the subgroup analysis. The results of pooled ORs, heterogeneity tests and publication bias tests for different analysis models are shown in Table 3 **(**Additional file 1: Figures S1 and S2).

**Table 3:** Main results of pooled ORs in meta-analysis of Vitamin D Receptor Gene Polymorphisms

Subgroup		Sample size	Test of association		Test of heterogeneity		Test of publication bias (Begg’s test)		Test of publication bias (Egger’s test)	
	Genetic model	Case/Control	OR	95% CI	I^2^ (%)	*P*	Z	*P*	T	*P*
FokI (rs2228570)										
Overall	Dominant model	3057 / 2852	1.06	0.94–1.19	45.7	0.02	−1.44	0.15	−1.09	0.29
	Recessive model	3057 / 2852	0.96	0.81–1.13	23.8	0.14	0.78	0.45	0.13	0.90
	Allelic model	3057 / 2852	1.08	0.93–1.26	66.6	≤0.001	0.63	0.52	0.46	0.65
	ff vs. FF	3057 / 2852	0.96	0.80–1.16	48.4	0.01	0.05	0.96	−0.63	0.54
	Ff vs.FF	3057 / 2852	1.06	0.93–1.19	26.4	0.16	−1.44	0.15	−1.33	0.20
European	Dominant model	2480 / 2202	1.10	0.97–1.26	41.7	0.08	−1.16	0.24	−0.62	0.55
	Recessive model	2480 / 2202	0.96	0.80–1.15	38.1	0.10	1.16	0.24	0.20	0.84
	Allelic model	2480 / 2202	1.04	0.90–1.20	56.5	0.01	0.27	0.78	−0.32	0.75
	ff vs. FF	2480 / 2202	1.00	0.75–1.33	42.8	0.07	0.27	0.78	−0.17	0.86
	Ff vs.FF	2480 / 2202	1.11	0.97–1.28	24.9	0.21	0.27	0.78	0.24	0.81
Asian	Dominant model	243 / 222	1.05	0.68–1.61	70.9	0.03	−0.52	0.60	−0.86	0.54
	Recessive model	243 / 222	1.27	0.21–7.61	57.9	0.12	−1	0.31	*	*
	Allelic model	243 / 222	1.06	0.46–2.45	80.9	≤0.001	−0.52	0.60	−0.36	0.77
	ff vs. FF	243 / 222	0.51	0.02–14.1	84	0.01	1	0.31	*	*
	Ff vs. FF	243 / 222	0.93	0.60–1.45	43.5	0.17	−0.52	0.60	−1.02	0.42
TaqI (rs731236)										
Overall	Dominant model	3758/3992	1.26	0.99–1.60	80.5	≤0.001	−0.13	0.89	0.38	0.71
	Recessive model	3758/3992	0.19	0.91–1.57	63	≤0.001	1.75	0.08	1.46	0.16
	Allelic model	3758/3992	1.16	0.94–1.42	87.2	≤0.001	−0.87	0.38	0.24	0.81
	tt vs. TT	3758/3992	1.26	0.93–1.71	65.9	≤0.001	0.54	0.58	0.98	0.34
	Tt vs.TT	3758/3992	1.27	**1.01–1.59**	74.5	≤0.001	−0.50	0.61	0.43	0.67
European	Dominant model	2785 / 2706	1.11	0.99–1.25	0	0.90	−0.80	0.42	0.08	0.94
	Recessive model	2785 / 2706	1.04	0.88–1.23	0	0.63	1.17	0.24	1.14	0.27
	Allelic model	2785 / 2706	1.06	0.97–1.15	0	0.76	−1.55	0.12	0.05	0.96
	tt vs. TT	2785 / 2706	1.11	0.92–1.33	0	0.82	0.63	0.52	0.98	0.34
	Tt vs.TT	2785 / 2706	1.12	0.99–1.27	0	0.79	−0.72	0.47	−0.08	0.94
Asian	Dominant model	453 / 471	1.53	0.34–6.95	95	≤0.001	−0.98	0.32	−0.24	0.82
	Recessive model	453 / 471	2.98	0.55–16.2	84.4	≤0.001	0	1	0.22	0.84
	Allelic model	453 / 471	1.43	0.40–5.13	96.9	≤0.001	−1.96	0.05	−0.59	0.59
	tt vs. TT	453 / 471	4.13	0.41–41.8	90.5	≤0.001	0	1	0.18	0.87
	Tt vs.TT	453 / 471	1.31	0.38–4.54	91.7	≤0.001	−0.98	0.32	−0.25	0.81
BsmI(rs1544410)										
	Dominant model	1793 / 1815	0.84	0.48–1.49	91.3	≤0.001	−0.24	0.80	1.81	0.09
	Recessive model	1793 / 1815	1.30	0.92–1.85	62.9	≤0.001	0.93	0.35	1.67	0.12
	Allelic model	1793 / 1815	1.10	0.89–1.37	69.7	≤0.001	0.18	0.85	0.23	0.82
	bb vs. BB	1793 / 1815	1.24	0.78–1.99	64.9	≤0.001	−1.95	0.05	−0.55	0.59
	Bb vs.BB	1793 / 1815	1.15	0.96–1.37	49.6	0.02	−1.46	0.14	−1.65	0.12
European	Dominant model	896 / 764	0.62	0.25–1.55	93.3	≤0.001	−0.19	0.85	1.50	0.2
	Recessive model	896 / 764	0.84	0.65–1.09	0	0.66	1.47	0.14	1.84	0.16
	Allelic model	896 / 764	0.99	0.85–1.16	0	0.51	−0.94	0.34	−1.45	0.22
	bb vs. BB	896 / 764	1.07	0.76–1.50	0	0.46	−1.96	0.05	−1.29	0.28
	Bb vs.BB	896 / 764	1.08	0.72–1.62	54.1	0.05	−0.94	0.34	−2.34	0.07
Asian	Dominant model	676 / 771	1.09	0.54–2.22	78.5	≤0.001	0	1	−0.30	0.78
	Recessive model	676 / 771	1.78	**1.08–2.93**	44.2	0.09	−1.35	0.17	−1.01	0.35
	Allelic model	676 / 771	1.28	0.81–2.02	79	≤0.001	−0.49	0.69	0.26	0.80
	bb vs. BB	676 / 771	1.50	0.46–4.88	76.3	≤0.001	−1.36	0.17	−0.97	0.43
	Bb vs.BB	676 / 771	1.08	0.59–1.96	66.9	0.01	−0.49	0.62	−0.12	0.91
ApaI (rs7975232)										
	Dominant model	2306 / 2669	1.04	0.82–1.31	58	≤0.001	−1.23	0.21	−1.14	0.26
	Recessive model	2306 / 2669	0.83	0.66–1.05	51	≤0.001	−0.58	0.55	−0.57	0.57
	Allelic model	2306 / 2669	0.94	0.80–1.10	68.2	≤0.001	−0.55	0.58	−1.02	0.32
	aa vs. AA	2306 / 2669	0.85	0.63–1.16	55.1	≤0.001	−0.78	0.43	−1.26	0.22
	Aa vs. AA	2306 / 2669	1.20	0.88–1.64	72.2	≤0.001	−0.97	0.33	−0.64	0.53
European	Dominant model	1366 / 1539	1.13	0.87–1.47	49.8	0.04	0	1	0.21	0.84
	Recessive model	1366 / 1539	1.01	0.78–1.33	38.7	0.11	−0.42	0.67	−0.49	0.64
	Allelic model	1366 / 1539	1.05	0.88–1.24	53.6	0.02	0	1	−0.56	0.59
	aa vs. AA	1366 / 1539	1.11	0.76–1.63	56.9	0.01	0.83	0.40	0.06	0.95
	Aa vs. AA	1366 / 1539	1.41	0.86–2.31	81.4	≤0.001	0.83	0.40	0.52	0.61
Asian	Dominant model	676 / 816	0.87	0.49–1.53	70.9	≤0.001	−1.73	0.08	−2.67	0.03
	Recessive model	676 / 816	0.61	**0.42–0.89**	40.4	0.11	0.99	0.32	0.64	0.54
	Allelic model	676 / 816	0.81	0.57–1.15	76.1	≤0.001	1.37	0.17	0.69	0.51
	aa vs. AA	676 / 816	0.52	**0.32–0.86**	28	0.20	0.25	0.80	0.37	0.72
	Aa vs. AA	676 / 816	1.03	0.59–1.79	64.5	≤0.001	−2.23	0.02	−2.17	0.07

The significant values are presented with boldface

### Meta-analysis for TaqI (rs731236) polymorphism and MS

**Fig. 2:**
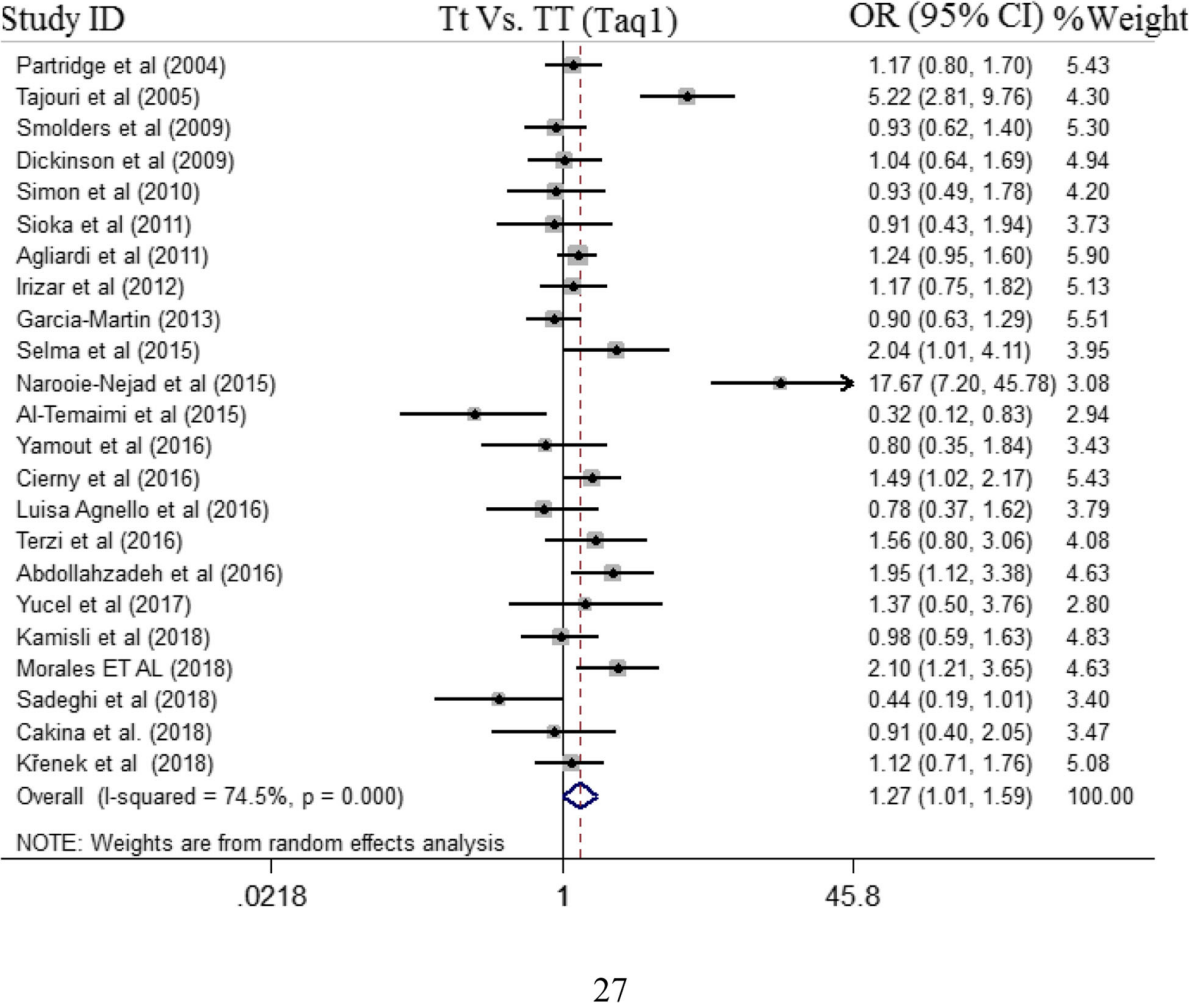
Pooled OR and 95% CI of individual studies and pooled data for the association between *TaqI* gene polymorphism and MS risk in Tt vs. TT Model (TaqI)

There were 23 case-control studies with 3758 cases and 3992 controls concerning TaqI polymorphism and MS risk. Among them, 13 studies were conducted in European countries [21, 22, 24, 34, 36, 37, 39, 40, 45–49], 5 studies in Asian countries [18, 41, 42, 50, 51], 2 studies in each Australian [17, 44] and American [43, 52] countries, and one study in Tunisia [20]. The TaqI polymorphism was demonstrated to be associated with MS risk under heterozygote contrast (OR = 1.27, 95%CI = 1.01–1.59, random effect) (Fig. 2), whilst no significant association was detected across other genotype models (Table 3). In addition, the pooled results of subgroup analysis decline presence of significant association under all defined genetic model **(**Additional file 1: Figures S3 and S4). Groups with less than three studies were removed from subgroup analysis. The results of pooled ORs, heterogeneity tests and publication bias tests for different analysis models are shown in Table 3.

### Meta-analysis for BsmI (rs1544410) polymorphism and MS

**Fig. 3:**
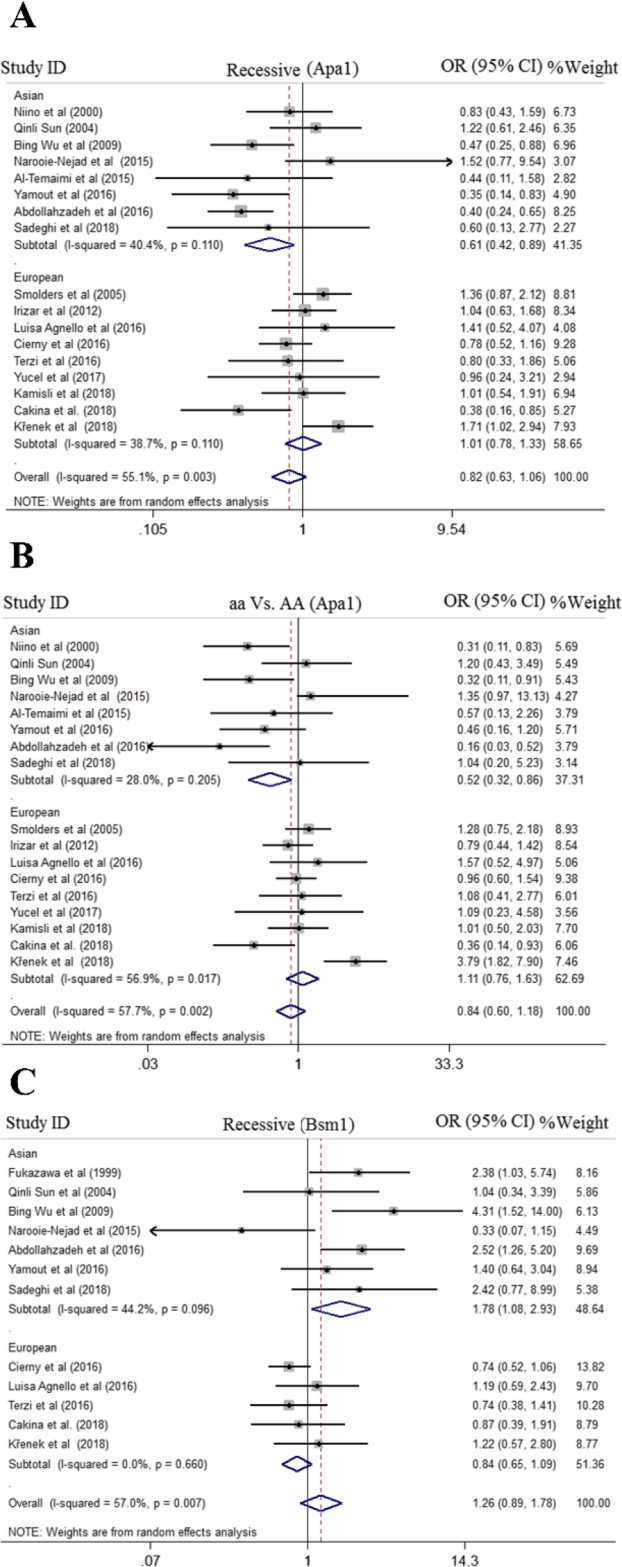
Pooled odds ratio (OR)) and 95% confidence interval of individual studies and pooled data for the association between *BsmI, ApaI* gene polymorphism and MS risk in different ethnicity subgroups and overall populations for A; Recessive Model (*ApaI*), B; aa vs. AA Model (*ApaI*), and C; Recessive Model *(BsmI*)

After searching databases, finally 16 case-control studies with 1793 cases and 1815 controls subjects included to examine association between BsmI polymorphism and MS risk. Among 16 studies, six studies were performed in Europe [21, 22, 36, 40, 48, 49], eight studies in Asia [18, 41, 50, 51, 53–55], and only two studies in America continent [43, 52]. The pooled results demonstrate no significant association between BsmI polymorphism and MS risk under all genetic models, but subgroup analysis revealed that BsmI polymorphism across recessive model increased the risk of MS in Asian population (OR = 1.78, 95%CI = 1.01–2.93, random effect) compared to European population (OR = 0.84, 95%CI = 0.66–1.06, random effect) (Fig. 3). The results of pooled ORs, heterogeneity tests and publication bias tests for different analysis models are shown in Table 3 **(**Additional file 1: Figures S5 and S6).

### Meta-analysis for Apa1 (rs7975232) polymorphism and MS

For quantitative synthesis of association between ApaI polymorphism and MS risk, 20 case-control studies with 2306 cases and 2669 controls were identified to be eligible. Overall, nine studies in Europe [21, 36, 37, 39, 40, 45, 46, 48, 49], eight studies in Asia [18, 41, 42, 50, 51, 54–56], and one study in Africa [20], America [43] and Australia [17] were identified. There was no evidence of association between ApaI polymorphism and MS risk in the pooled results. However, subgroup analysis detected significant association between presence of ApaI SNP and risk of MS under recessive model (OR = 0.61, 95%CI = 0.42–0.89, random effect) and homozygote model (OR = 0.52, 95%CI = 0.32–0.86, random effect) in Asian population in comparison with European population (OR = 1.01, 95%CI = 0.78–1.33, recessive model) and (OR = 1.11, 95%CI = 0.76–1.63, homozygote model) (Fig. 3). The results of pooled ORs, heterogeneity tests, and publication bias tests for different analysis models are shown in Table 3 **(**Additional file 1: Figures S7 and S8).

### Evaluation of heterogeneity

Significant heterogeneity existed for FokI, TaqI, BsmI, and ApaI polymorphism in all of the genetic models. Furthermore, in subgroup analysis, there was a significant heterogeneity for studies carried out in Asian and European countries (Table 3).

### Publication bias and Sensitivity analysis

**Fig. 4:**
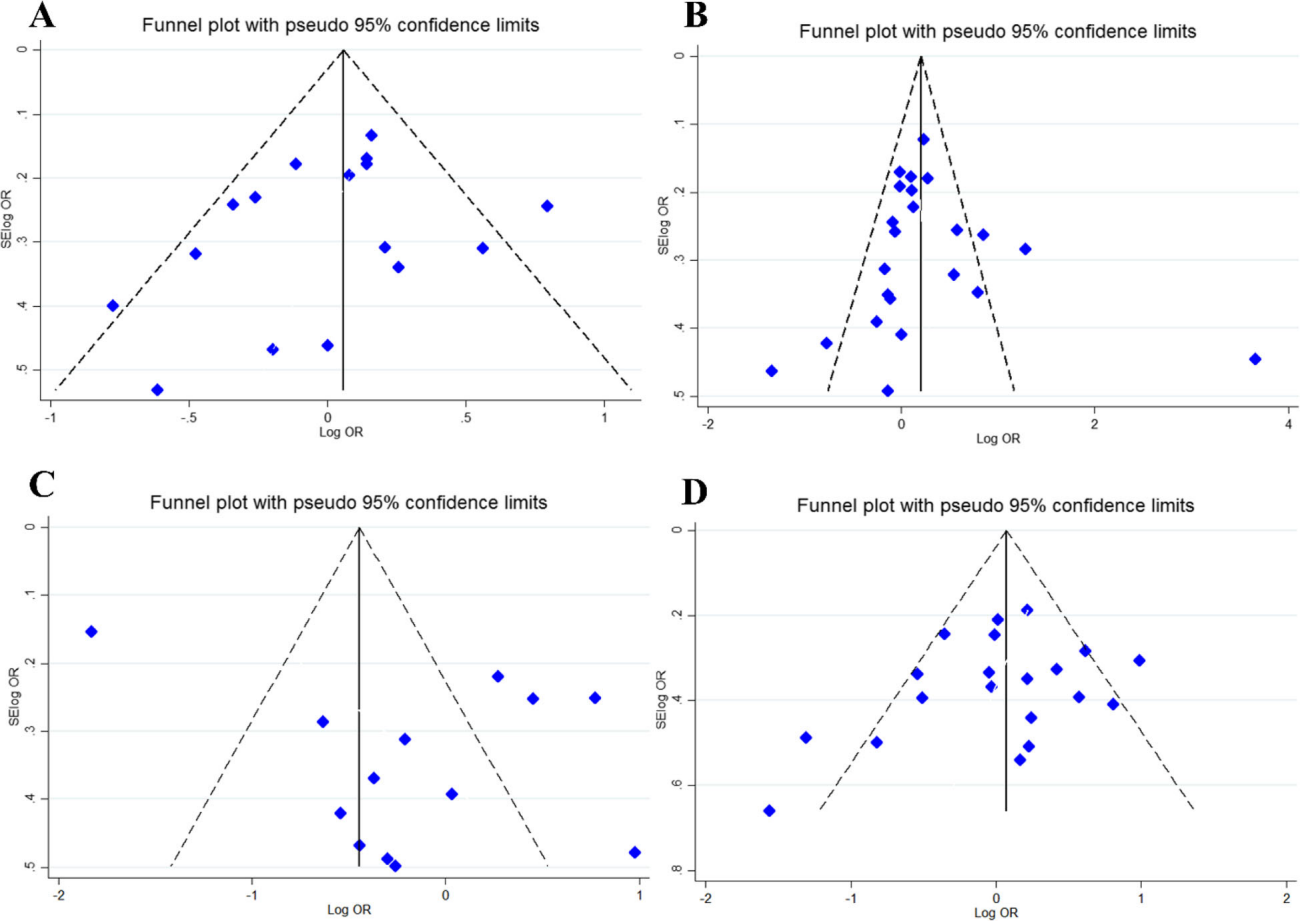
Begg’s funnel plot for publication bias test. **a**; Dominant Model *FokI* .**b**; Dominant Model *TaqI*. **c**; Dominant Model *BsmI.* **d**; Dominant Model *ApaI*. Each point represents a separate study for the indicated association

**Fig. 5:**
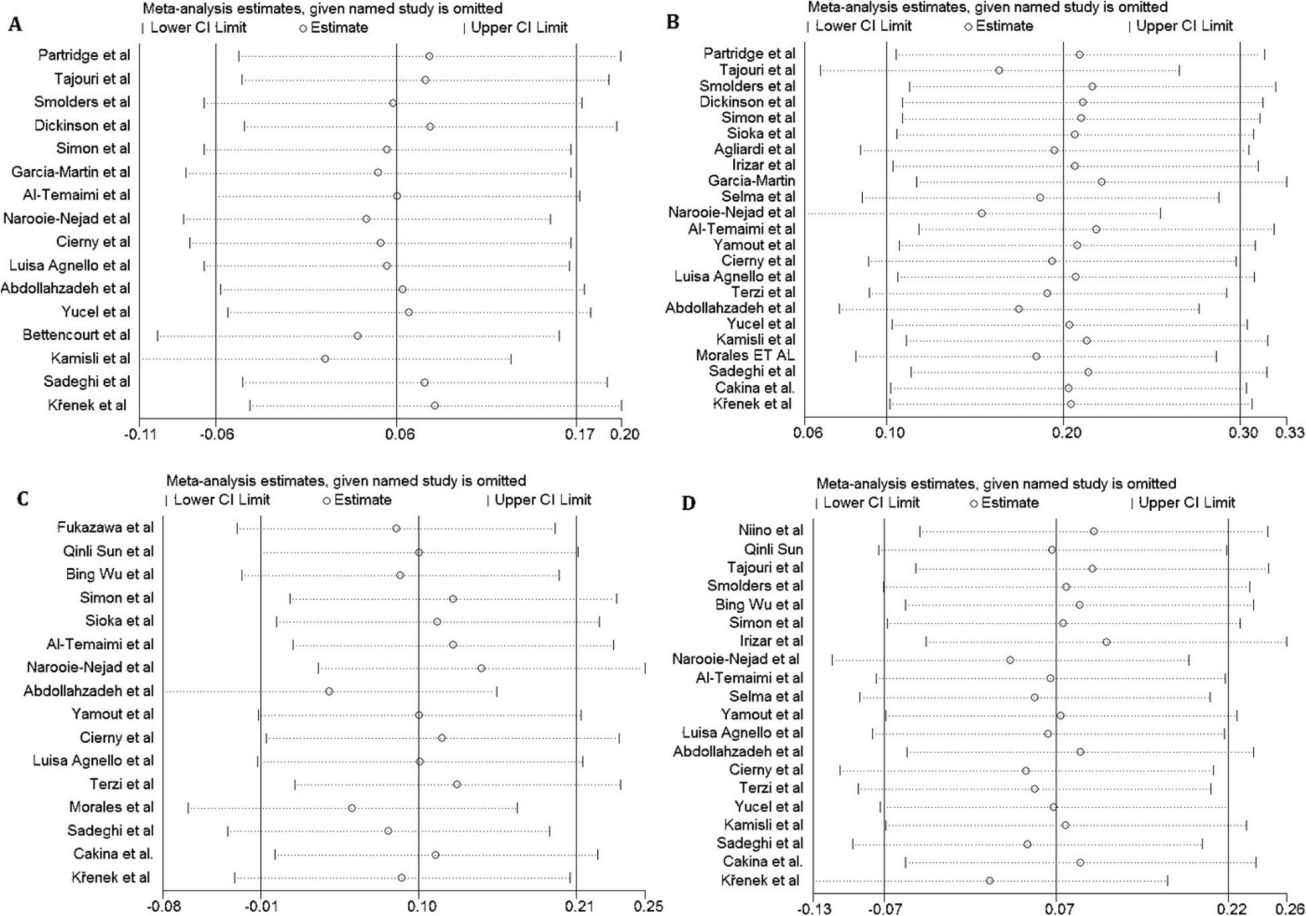
Sensitivity analysis in present meta-analysis investigates the single nucleotide polymorphisms of Vitamin D Receptor contribute to risk for multiple sclerosis susceptibility (A, FokI; B, TaqI; C, BsmI; D, ApaI)

Publication bias was estimated using funnel plot, Begg’s and Egger’s tests. No evidence of publication bias was seen for all four SNP and subgroup analysis under all genetic models. Additionally, the shape of the funnel plot appeared to be symmetrical, indicating that there was no significant publication bias (Fig. 4).The impact of individual study on pooled OR was estimated by sequential omission of each studies which results showed that no individual study significantly affected the pooled ORs under any genetic models of the VDR SNPs (Fig. 5).

## Discussion

*VDR* gene, as a pleiotropic gene, has been shown to be associated with several diseases. In the previous studies, the relationship between VDR gene single nucleotide polymorphisms and autoimmune disorders was evaluated in several meta-analyses. The study of Feng et al. [57]. described that TaqI or BsmI polymorphism in *VDR* gene was significantly connected with autoimmune thyroid diseases. Mao et al. [58]. represented that the BsmI B allele may act as a risk factor for onset of systemic lupus erythematous (SLE) among Asians and overall populations and also the FokI FF genotype act as a potential risk factor for SLE predisposition in Asians. Furthermore, Tizaoui et al. [59] showed that the *VDR* gene TaqI and FokI polymorphisms may increase the risk of Rheumatoid arthritis (RA) in European populations. And finally, Wang et al. [60] reported that the ApaI and BsmI polymorphisms were related with elevated susceptibility to type 1 diabetes (T1D) in Asian populations. Collectively, it could be assumed that *VDR* gene polymorphisms act as a potential risk factor in development or progression of autoimmune disorders.

Although four meta-analyses have been conducted in the past 10 years to evaluate the relationship between the *VDR* gene polymorphisms and MS, these findings were inconclusive due to the variations of the literature and selected databases. Hence, for resolving these inconsistencies, and to decrease the heterogeneity and the probability of random errors, we set out an updated meta-analysis. In this study, 30 studies met the inclusion criteria and were included for quantitative synthesis. No evidence of publication bias was observed for all four SNP in subgroup analysis and overall populations under five genetic models. Regarding the essential role of genetic factors in the pathogenesis of MS, we categorized our results according to ethnicity. Our meta-analysis revealed that BsmI, ApaI, and TaqI polymorphisms may play a significant role in the pathogenesis of MS in overall and Asian population. The results of this study indicated that TaqI polymorphism was associated with MS susceptibility under heterozygote contrast in overall population.

Subgroup analysis based on continent rejected any association between *VDR* gene polymorphisms and the MS risk in European population. However, a significant association between BsmI and ApaI polymorphisms and MS susceptibility was detected in Asian population. BsmI polymorphism was associated with 64% increased risk of MS under recessive model in Asian populations. On the other hand, ApaI polymorphism was associated with 37.5 and 34.5% decreased risk of MS under recessive model and aa vs. AA model in Asian populations, respectively. The possible reason of the conflicting results among Asian, European, and overall population could be related to environmental factors the individuals exposed to and different genetic backgrounds, which may have disproportionate effects on MS risk.

These findings are inconsistent with the results of the Huang et al. [23] and Garcia-Martin et al. [24] studies. In the study of Huang et al.., 11 case-control studies with 2599 cases and 2816 controls were included for assessing the association between *VDR* gene polymorphisms and the MS susceptibility, but no significant association was found. Another study by Garcia-Martin et al. that analyzed ten studies with 2944 MS patients and 3166 healthy subjects, reported that TaqI and FokI polymorphisms were not associated with the MS risk. In accordance with our study, the study of Zhang et al. [25]. and Tizaoui et al. [26]. showed a significant association between *VDR* gene polymorphisms and the MS susceptibility. However, there are some obvious differences in the findings of these studies in comparison with our study. Meta-analysis of Tizaoui et al.. reported an association of the FokI FF and ApaI AA genotypes with an elevated susceptibility of MS in a total of 3300 MS patients and 3194 healthy subjects from 13 case-control studies. In contrast, our analysis, consisting of 20 case-control studies, showed that ApaI polymorphism was associated with decreased risk of MS in Asian populations. In addition, the study of Zhang et al. reported that the A allele was related with the onset of disease in Asian populations. Nevertheless, the sensitivity analysis, by removing the studies not in HWE, rejected any association between the A allele and risk of MS, which was dissimilar to the results of the non-sensitivity analysis. Moreover, they failed to find any association between TaqI, BsmI, and ApaI polymorphisms and MS susceptibility in overall populations, Asians, and Caucasians. The main reasons that *VDR* gene polymorphism plays a diverse function across different studies or in different ethnic populations may be due to the following hints. Firstly, in many cases, controls in included studies deviated from HWE. Secondly, the differences in the ethnic contextual characteristics of the patients may be an important factor for these variations. Thirdly, *VDR* SNPs were suggested to be related with the basal levels of 1,25(OH)2D3 and vitamin D structure and function [61], which in turn could influence MS predisposition. Finally, MS is regarded to be a polygenic disorder, and therefore it is expected that various gene loci are interacted in the pathogenesis of MS.

Several epidemiological studies have strongly proposed that vitamin D insufficiency and sunshine contributes to MS risk in temperate countries. Vitamin D sufficiency and insufficiency could be a protective and risk factor, respectively, among many other factors, and may be constantly regulating the global MS susceptibility from the mother’s pregnancy to adulthood. The main role of vitamin D in MS seems to be immunomodulatory, affecting the different groups of T and B cells in the general immune system, however, neurotrophic and neuroprotectant mechanisms could also be applied at the central nervous system (CNS) [62, 63]. Interestingly, in clinical setting, correction of hypovitaminosis D through recommending oral D3 supplements resulted in raises in 25(OH)D levels in serum, which were correlated with reductions in annualized relapse-rate (ARR) in relapsing-remitting MS (RRMS) [64]. The disease activity is generally improved with higher 25(OH)D level. Rotstein et al.. reported that in MS patients under fingolimod (FTY) therapy, higher 25(OH)D level was related to longer survival for the combined endpoint and for relapses [65]. A recent randomized clinical trial reveled a potential therapeutic effect of cholecalciferol in RRMS patients with low serum 25OHD level, which already treated with interferon beta-1a [66]. Furthermore, comprehensive systematic review by Dörr et al.. based on many line of data, including preclinical investigations, association studies, epidemiologic data, and animal studies on vitamin D status and disease activity, implies that higher serum level of vitamin D are beneficial in terms of the susceptibility to MS as well as the further course of the disease in patients with established MS [67]. In the earliest phase of disease, lower levels of 25-hydroxyvitamin D correlates with higher disease activity, however, lower 25(OH)D3 levels hardly affects patients in terms of clinical presentations, implying that low 25(OH)D3 concentrations are rather a susceptibility factor for than an outcome of MS; Since the bioavailable vitamin D concentration did not differ between the MS patients and healthy subjects, the main mechanism underlying the association of vitamin D and MS does not seem to be linked with decreased vitamin D bioavailability [68]. Despite all that has been discussed, a meta-analysis by Zheng and colleagues reported that vitamin D had no therapeutic effect on ARR and Expanded Disability Status Scale (EDSS) score in the patients with MS [69].

Permutations and combinations of common variants account as a predisposition factors in the etiology of several complex diseases. Variations of DNA sequence like SNPs exert modest biological impacts [11]. Three polymorphisms of VDR gene, including TaqI, ApaI, and BsmI do not influence the structure of VDR protein. Their affect may be associated with alterations in translation efficiency and/or stability of the RNA. On the other hand, the FokI polymorphism has been related to changes in both transcriptional activity and VDR protein structure [70]. The wild-type short transcript of FokI is related with the elevated transcriptional activity [70]. One potential exception is differential effect of the FokI polymorphism on the immune system [16]. Our data suggested that the ApaI polymorphism has a significant functional effect on MS. Furthermore, the TaqI polymorphism was associated with MS risk. However, some other factors that were not examined in the current meta-analysis might affect the TaqI expression. At this point, the expression and function of VDR in transactivating target genes are indicated by environment, genetics, and ethnicity due to its complex interactions [71]. Thus far, three essential environmental risk factors for MS have been determined: vitamin D insufficiency, cigarette smoking, and Epstein–Barr virus infection [72, 73]. Moreover, sun exposure interacts with VDR gene functional variants in childhood to affect MS predisposition.

Some limitations of this meta-analysis should be considered. First, inaccessibility to the original data of the included studies restricted our further assessment of potential interactions, since the interactions between and even various polymorphic region of the same gene may affect the risk. Moreover, this study was solely focused on the articles published in the English language. We detected significant heterogeneity in all of the genetic models, which could be derived by various factors, such as variations in ethnicities. In the current study, ethnicities were Caucasians from Asians, Caucasians from Europe and Australia. Also, heterogeneity may be created by publication year of included studies, which extended between 1999 and 2018. There are several other possible reasons which may be regarded as a source of heterogeneity. Firstly, the criteria of MS diagnosis are inconsistent between studies. While some of them employed Poser’s criteria, other studies used McDonald’s criteria for MS diagnosis. Secondly, gender may act as a potential source for heterogeneity. Although both male and female subjects were enrolled in most studies, two studies were not sex-matched and one study only included women subjects [35, 43, 45]. Thirdly, genotyping methods were not consistent. While half of the included studies used PCR-RFLP, approximately the other half employed TaqMan assay and one study used PCR-SPP. Fourthly, geographical and ethnic factors may also participate in heterogeneity, because studies with the same ethnic source were accompanied in various geographical regions.

The results from the studies examined in this met-analysis should be interpreted with cautious for some reasons. Our findings suggest that, to afford accurate estimates of the relation between VDR polymorphisms and MS risk, several factors should be regarded. Although there are many functional VDR polymorphisms in the promoter region of the VDR gene, only four SNPs in the VDR gene have been evaluated. The interaction of the *MHC* genes with *VDR* gene have been demonstrated to be important in MS [74, 75]. Remarkably, various environmental factors may interact with VDR polymorphisms to alter MS susceptibility. The current meta-analysis could not assess all interactions between VDR polymorphisms and study characteristics because of insufficient data from the original publications.

## Conclusion

Taken all together, the current meta-analysis affords a comprehensive investigation of the available information for the association between the VDR polymorphisms and MS susceptibility. This meta-analysis of 30 case-control studies reveled a significant association between TaqI polymorphism and MS susceptibility. In subgroup analysis, BsmI polymorphism was associated with increased risk of MS in Asian populations. In addition, ApaI polymorphism was associated with decreased risk of MS in Asian populations. However, neither in overall population nor in subgroup analysis significant association between Fok1 (rs2228570) polymorphism and MS susceptibility was found. Future large-scale studies on gene–environment and gene–gene interactions are required to estimate related risk factors and assist early diagnosis of patients at high risk for MS.

## Supplementary information

**Additional file 1: Figure 1.** Forest plot of association between Fok1 gene Polymorphism and MS risk; Dominant model, Recessive model allelic model, ff VS. FF model, Ff vs FF model. **Figure 2.** Forest plot of pooled odds ratio (OR)) and 95% confidence interval of individual studies and pooled data for the association between Fok1 polymorphism and MS risk in different ethnicity subgroups and overall populations for Dominant model, Recessive model, allelic model, ff VS. FF model, Ff vs FF model. **Figure 3.** Forest plot of association between Taq1 gene Polymorphism and MS risk; Dominant model, Recessive model, allelic model, tt VS. TT model, Tt vs TT model. **Figure 4.** Forest plot of pooled odds ratio (OR)) and 95% confidence interval of individual studies and pooled data for the association between Taq1 polymorphism and MS risk in different ethnicity subgroups and overall populations for Dominant model, Recessive model, allelic model, tt vs TT model, Tt vs TT model. **Figure 5.** Forest plot of association between Bsm1 gene Polymorphism and MS risk; Dominant model, Recessive model, allelic model, bb VS. BB model, Bb vs BB model. **Figure 6.** Forest plot of pooled odds ratio (OR)) and 95% confidence interval of individual studies and pooled data for the association between Bsm1polymorphism and MS risk in different ethnicity subgroups and overall populations for Dominant model, Recessive model allelic model, bb VS. BB model, Bb vs BB model. **Figure 7.** Forest plot of association between Apa1 gene Polymorphism and MS risk; Dominant model, Recessive model, allelic model, aa VS. AA model, Aa vs AA model. **Figure 8.** Forest plot of pooled odds ratio (OR)) and 95% confidence interval of individual studies and pooled data for the association between Apa1 polymorphism and MS risk in different ethnicity subgroups and overall populations for Dominant model, Recessive model, allelic model, aa VS. AA model, Aa vs AA model.

## Data Availability

The datasets used and/or analysed during the current study are available from the corresponding author on reasonable request.

## Abbreviations

CI: Confidence Interval
CNS: Central nervous system
HLA: Human leukocyte antigen
LD: Linkage disequilibrium
MHC-II: Major histocompatibility complex II
MS: Multiple sclerosis
OR: Odd Ratio
PCR-SPP: Polymerase chain reaction - Sequence Specific
PRISMA: Preferred Reporting Items for Systematic reviews and Meta-Analyses
RFLP: Restriction fragment length polymorphisms
SLE: Systemic lupus erythematosus
SNP: Single Nucleotide Polymorphisms
T1D: Type 1 diabetes
UTR: Untranslated region
VDR: Vitamin D receptor polymorphism

## References

[1] Imani D (2018). Association of nod-like receptor protein-3 single nucleotide gene polymorphisms and expression with the susceptibility to relapsing–remitting multiple sclerosis. Int J Immunogenet.

[2] Emamnejad R (2019). Circulating mesenchymal stem cells, stromal derived factor (SDF)-1 and IP-10 levels increased in clinically active multiple sclerosis patients but not in clinically stable patients treated with beta interferon. Mult Scler Relat Disord.

[3] Longo DL, Reich DS, Lucchinetti CF, Calabresi PA (2018). N Engl J Med.

[4] Krieger SC (2016). The topographical model of multiple sclerosis: a dynamic visualization of disease course. Neurology-Neuroimmunol Neuroinflamm.

[5] McFarland HF, Martin R (2007). Multiple sclerosis: a complicated picture of autoimmunity. Nat Immunol.

[6] Adorini L, Penna G (2008). Control of autoimmune diseases by the vitamin D endocrine system. Nat Rev Rheumatol.

[7] DeLuca HF (2004). Overview of general physiologic features and functions of vitamin D. Am J Clin Nutr.

[8] Gorman S (2007). Topically applied 1, 25-dihydroxyvitamin D3 enhances the suppressive activity of CD4+ CD25+ cells in the draining lymph nodes. J Immunol.

[9] Smolders J (2008). Association of vitamin D metabolite levels with relapse rate and disability in multiple sclerosis. Mult Scler J.

[10] Runia TF (2012). Lower serum vitamin D levels are associated with a higher relapse risk in multiple sclerosis. Neurol.

[11] Uitterlinden AG (2004). Genetics and biology of vitamin D receptor polymorphisms. Gene.

[12] Zmuda JM, Cauley JA, Ferrell RE (2000). Molecular epidemiology of vitamin D receptor gene variants. Epidemiol Rev.

[13] Makoui MH, et al. Vitamin D receptor gene polymorphism and susceptibility to asthma: meta-analysis based on 17 case-control studies. Ann Allergy Asthma Immunol. 2019;124(1):57–69. 10.1016/j.anai.2019.10.014 31654764

[14] Ručević I (2009). Vitamin D endocrine system and psoriasis vulgaris-review of the literature. Acta Dermatovenerol Croat.

[15] Saccone D, Asani F, Bornman L (2015). Regulation of the vitamin D receptor gene by environment, genetics and epigenetics. Gene.

[16] van Etten E (2007). The vitamin D receptor gene FokI polymorphism: functional impact on the immune system. Eur J Immunol.

[17] Tajouri L (2005). Variation in the vitamin D receptor gene is associated with multiple sclerosis in an Australian population. J Neurogenet.

[18] Al-Temaimi RA (2015). The association of vitamin D receptor polymorphisms with multiple sclerosis in a case-control study from Kuwait. PLoS One.

[19] Narooie-Nejad M (2015). Positive association of vitamin D receptor gene variations with multiple sclerosis in South East Iranian population. BioMed Res Int.

[20] Ben-Selma W (2015). Age-and gender-specific effects on VDR gene polymorphisms and risk of the development of multiple sclerosis in Tunisians: a preliminary study. Int J Immunogenet.

[21] Čierny D (2015). FokI vitamin D receptor gene polymorphism in association with multiple sclerosis risk and disability progression in Slovaks. Neurol Res.

[22] Sioka C (2011). Vitamin D receptor gene polymorphisms in multiple sclerosis patients in Northwest Greece. J Negat Results Biomed.

[23] Huang J, Xie Z-F (2012). Polymorphisms in the vitamin D receptor gene and multiple sclerosis risk: a meta-analysis of case–control studies. J Neurol Sci.

[24] García-Martín E (2013). Vitamin D3 receptor (VDR) gene rs2228570 (Fok1) and rs731236 (Taq1) variants are not associated with the risk for multiple sclerosis: results of a new study and a meta-analysis. PLoS One.

[25] Zhang Y-J (2018). Association between VDR polymorphisms and multiple sclerosis: systematic review and updated meta-analysis of case-control studies. Neurol Sci.

[26] Tizaoui K (2015). Association between vitamin D receptor polymorphisms and multiple sclerosis: systematic review and meta-analysis of case–control studies. Cell Mol Immunol.

[27] Moher D (2009). Preferred reporting items for systematic reviews and meta-analyses: the PRISMA statement. Ann Intern Med.

[28] Stang A (2010). Critical evaluation of the Newcastle-Ottawa scale for the assessment of the quality of nonrandomized studies in meta-analyses. Eur J Epidemiol.

[29] Huedo-Medina TB (2006). Assessing heterogeneity in meta-analysis: Q statistic or I2 index?. Psychol Methods.

[30] Mantel N, Haenszel W (1959). Statistical aspects of the analysis of data from retrospective studies of disease. J Natl Cancer Inst.

[31] DerSimonian R, Laird N (1986). Meta-analysis in clinical trials control Clin trials 7: 177–188.

[32] Egger M (1997). Bias in meta-analysis detected by a simple, graphical test. BMJ.

[33] Begg Colin B., Mazumdar Madhuchhanda (1994). Operating Characteristics of a Rank Correlation Test for Publication Bias. Biometrics.

[34] Partridge J (2004). Susceptibility and outcome in MS: associations with polymorphisms in pigmentation-related genes. Neurol.

[35] Smolders J (2009). Fok-I vitamin D receptor gene polymorphism (rs10735810) and vitamin D metabolism in multiple sclerosis. J Neuroimmunol.

[36] Agnello L (2016). Vitamin D receptor polymorphisms and 25-hydroxyvitamin D in a group of Sicilian multiple sclerosis patients. Neurol Sci.

[37] Yucel FE (2018). Analysis of vitamin D receptor polymorphisms in patients with familial multiple sclerosis. Med Arch.

[38] Bettencourt A (2017). The vitamin D receptor gene FokI polymorphism and multiple sclerosis in a northern Portuguese population. J Neuroimmunol.

[39] Kamisli O (2018). The association between vitamin D receptor polymorphisms and multiple sclerosis in a Turkish population. Mult Scler Relat Dis.

[40] Křenek P, et al. The Impact of Five VDR Polymorphisms on Multiple Sclerosis Risk and Progression: a Case-Control and Genotype-Phenotype Study. J Mol Neurosci. 2018:1–8. 10.1007/s12031-018-1034-1 29589202

[41] Abdollahzadeh R (2016). Predisposing role of vitamin D receptor (VDR) polymorphisms in the development of multiple sclerosis: a case-control study. J Neurol Sci.

[42] SADEGHI A, et al. The BsmI, FokI, ApaI and TaqI Polymorphisms in Vitamin D Receptor Gene in Iranian Multiple Sclerosis Patients: A Case-Control Study. J iranian clin Res. 2015;3:28–32.

[43] Simon KC (2010). Polymorphisms in vitamin D metabolism related genes and risk of multiple sclerosis. Mult Scler J.

[44] Dickinson JL (2009). Past environmental sun exposure and risk of multiple sclerosis: a role for the Cdx-2 vitamin D receptor variant in this interaction. Mult Scler J.

[45] Smolders J (2009). Association study on two vitamin D receptor gene polymorphisms and vitamin D metabolites in multiple sclerosis. Ann N Y Acad Sci.

[46] Irizar H (2012). HLA-DRB1* 15: 01 and multiple sclerosis: a female association?. Mult Scler J.

[47] Agliardi C (2011). Vitamin D receptor (VDR) gene SNPs influence VDR expression and modulate protection from multiple sclerosis in HLA-DRB1* 15-positive individuals. Brain Behav Immun.

[48] Cakina S (2018). Vitamin D receptor gene polymorphisms in multiple sclerosis disease: a case-control study. Rev Romana De Med De Lab.

[49] Terzi, M., et al. Vitamin D Receptor Gene Polymorphism in Patients with Multiple Sclerosis. in *MULTIPLE SCLEROSIS JOURNAL*. 2018. SAGE PUBLICATIONS LTD 1 OLIVERS YARD, 55 CITY ROAD, LONDON EC1Y 1SP, ENGLAND.2018. 24(7) pp. 61–62.

[50] Narooie-Nejad M (2015). Vitamin D receptor gene polymorphism and the risk of multiple sclerosis in south eastern of Iran. J Mol Neurosci.

[51] Yamout B (2016). Vitamin D receptor biochemical and genetic profiling and HLA-class II genotyping among Lebanese with multiple sclerosis—a pilot study. J Neuroimmunol.

[52] Bermúdez-Morales VH (2017). Vitamin D receptor gene polymorphisms are associated with multiple sclerosis in Mexican adults. J Neuroimmunol.

[53] Fukazawa T (1999). Association of vitamin D receptor gene polymorphism with multiple sclerosis in Japanese. J Neurol Sci.

[54] Bing WHL, Tao W (2009). Association of vitamin D receptor gene polymorphism with multiple sclerosis. Henan Med Res.

[55] Qinlin SRX, Yinhua W, et al. Association of vitamin D receptor gene polymorphism with multiple sclerosis. Chin J Tissue Eng Res. 2004.

[56] Niino M (2000). Vitamin D receptor gene polymorphism in multiple sclerosis and the association with HLA class II alleles. J Neurol Sci.

[57] Feng Ming, Li Hua, Chen Su-Fang, Li Wei-Fang, Zhang Fang-Bin (2012). Polymorphisms in the vitamin D receptor gene and risk of autoimmune thyroid diseases: a meta-analysis. Endocrine.

[58] Mao S, Huang S (2014). Association between vitamin D receptor gene BsmI, FokI, ApaI and TaqI polymorphisms and the risk of systemic lupus erythematosus: a meta-analysis. Rheumatol Int.

[59] Tizaoui K, Hamzaoui K (2015). Association between VDR polymorphisms and rheumatoid arthritis disease: systematic review and updated meta-analysis of case–control studies. Immunobiol.

[60] Wang G, Kuanfeng X, Yang T (2015). Associations between polymorphisms of vitamin D receptor gene and type 1 diabetes susceptibility: a meta-analysis. Chin J Diab.

[61] Morán-Auth Y, Penna-Martinez M, Badenhoop K (2015). VDR FokI polymorphism is associated with a reduced T-helper cell population under vitamin D stimulation in type 1 diabetes patients. J Steroid Biochem Mol Biol.

[62] Pierrot-Deseilligny C, Souberbielle J-C (2017). Vitamin D and multiple sclerosis: an update. Mult Scler Relat Dis.

[63] Koduah P, Paul F, Dörr J-M (2017). Vitamin D in the prevention, prediction and treatment of neurodegenerative and neuroinflammatory diseases. Epma J.

[64] Laursen JH (2016). Vitamin D supplementation reduces relapse rate in relapsing-remitting multiple sclerosis patients treated with natalizumab. Mult Scler Relat Dis.

[65] Rotstein DL (2015). Effect of vitamin D on MS activity by disease-modifying therapy class. Neurol-Neuroimmunol Neuroinflamm.

[66] Camu W (2019). Cholecalciferol in relapsing-remitting MS: a randomized clinical trial (CHOLINE). Neurol-Neuroimmunol Neuroinflamm.

[67] Dörr J, Döring A, Paul F (2013). Can we prevent or treat multiple sclerosis by individualised vitamin D supply?. Epma J.

[68] Behrens JR (2016). Low 25-hydroxyvitamin D, but not the bioavailable fraction of 25-hydroxyvitamin D, is a risk factor for multiple sclerosis. Eur J Neurol.

[69] Zheng C (2018). The efficacy of vitamin D in multiple sclerosis: a meta-analysis. Mult Scler Relat Dis.

[70] Jurutka PW (2000). The polymorphic N terminus in human vitamin D receptor isoforms influences transcriptional activity by modulating interaction with transcription factor IIB. Mol Endocrinol.

[71] O’Gorman C, Lucas R, Taylor B (2012). Environmental risk factors for multiple sclerosis: a review with a focus on molecular mechanisms. Int J Mol Sci.

[72] Pierrot-Deseilligny C, Souberbielle J-C (2013). Contribution of vitamin D insufficiency to the pathogenesis of multiple sclerosis. Ther Adv Neurol Disord.

[73] Canbay C (2010). The essential environmental cause of multiple sclerosis disease. Prog Electromagn Res.

[74] Handunnetthi L, Ramagopalan SV, Ebers GC (2010). Multiple sclerosis, vitamin D, and HLA-DRB1* 15. Neurol.

[75] Ramagopalan SV (2009). Expression of the multiple sclerosis-associated MHC class II allele HLA-DRB1* 1501 is regulated by vitamin D. PLoS Genet.

