# Supplementary material for: Association between vitamin D receptor (VDR) polymorphisms and the risk of multiple sclerosis (MS): an updated meta-analysis
Source: BMC Neurol. 2019 Dec 26;19:339. doi: 10.1186/s12883-019-1577-y (PMC6933912; doi:10.1186/s12883-019-1577-y)
Supplement: Supplementary file 1 — Additional file 1: Figure 1. Forest plot of association between Fok1 gene Polymorphism and MS risk; Dominant model, Recessive model allelic model, ff VS. FF model, Ff vs FF model. Figure 2. Forest plot of pooled odds ratio (OR)) and 95% confidence interval of individual studies and pooled data for the association between Fok1 polymorphism and MS risk in different ethnicity subgroups and overall populations for Dominant model, Recessive model, allelic model, ff VS. FF model, Ff vs FF model. Figure 3. Forest plot of association between Taq1 gene Polymorphism and MS risk; Dominant model, Recessive model, allelic model, tt VS. TT model, Tt vs TT model. Figure 4. Forest plot of pooled odds ratio (OR)) and 95% confidence interval of individual studies and pooled data for the association between Taq1 polymorphism and MS risk in different ethnicity subgroups and overall populations for Dominant model, Recessive model, allelic model, tt vs TT model, Tt vs TT model. Figure 5. Forest plot of association between Bsm1 gene Polymorphism and MS risk; Dominant model, Recessive model, allelic model, bb VS. BB model, Bb vs BB model. Figure 6. Forest plot of pooled odds ratio (OR)) and 95% confidence interval of individual studies and pooled data for the association between Bsm1polymorphism and MS risk in different ethnicity subgroups and overall populations for Dominant model, Recessive model allelic model, bb VS. BB model, Bb vs BB model. Figure 7. Forest plot of association between Apa1 gene Polymorphism and MS risk; Dominant model, Recessive model, allelic model, aa VS. AA model, Aa vs AA model. Figure 8. Forest plot of pooled odds ratio (OR)) and 95% confidence interval of individual studies and pooled data for the association between Apa1 polymorphism and MS risk in different ethnicity subgroups and overall populations for Dominant model, Recessive model, allelic model, aa VS. AA model, Aa vs AA model. [file 12883_2019_1577_MOESM1_ESM.docx]

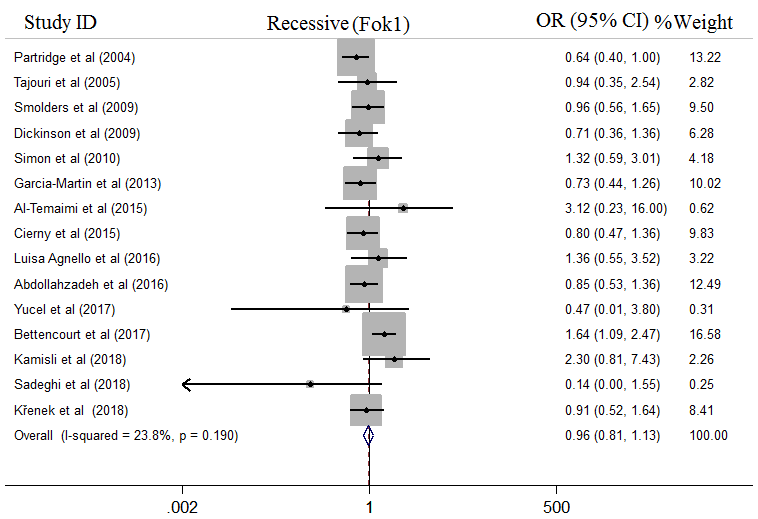

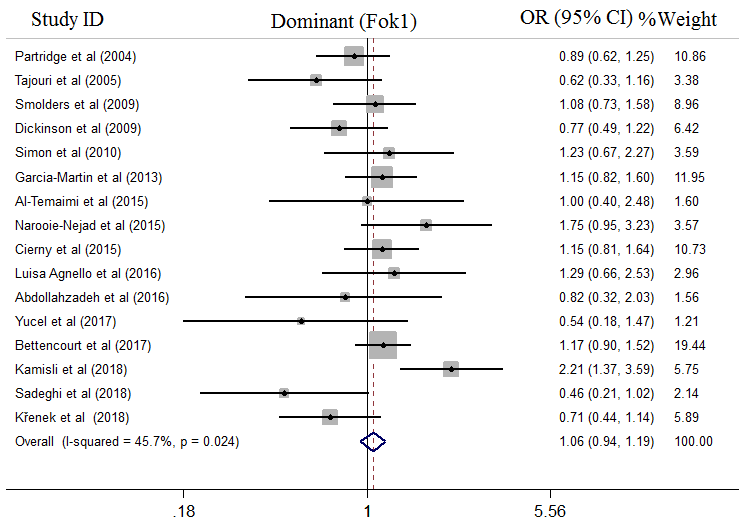


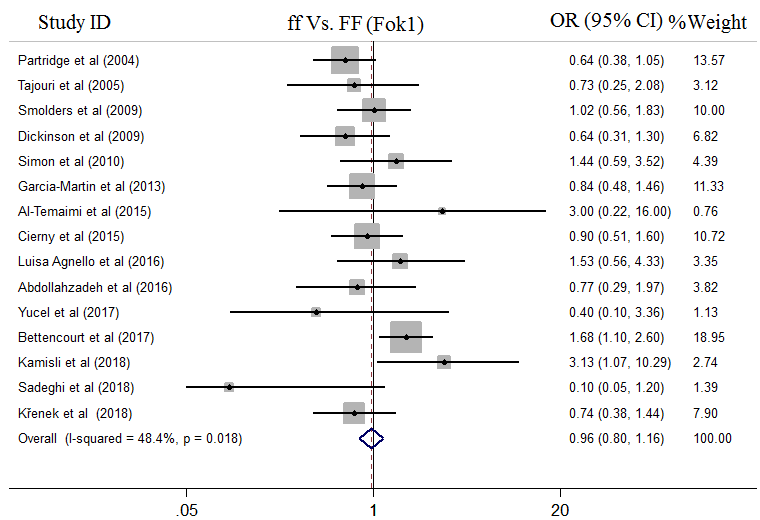

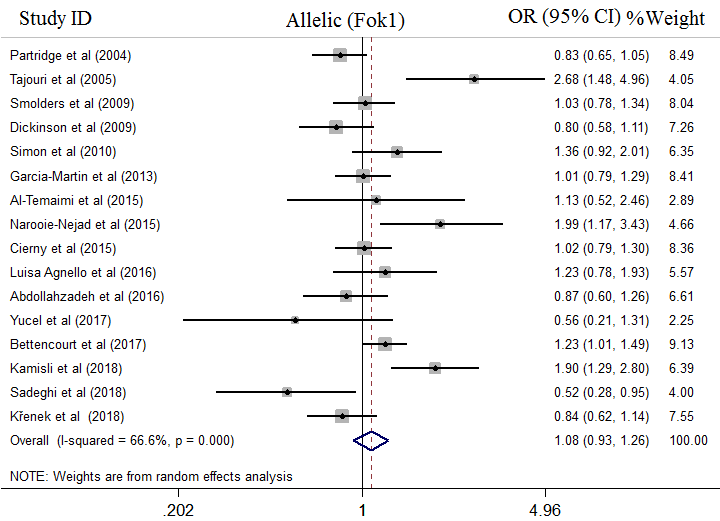


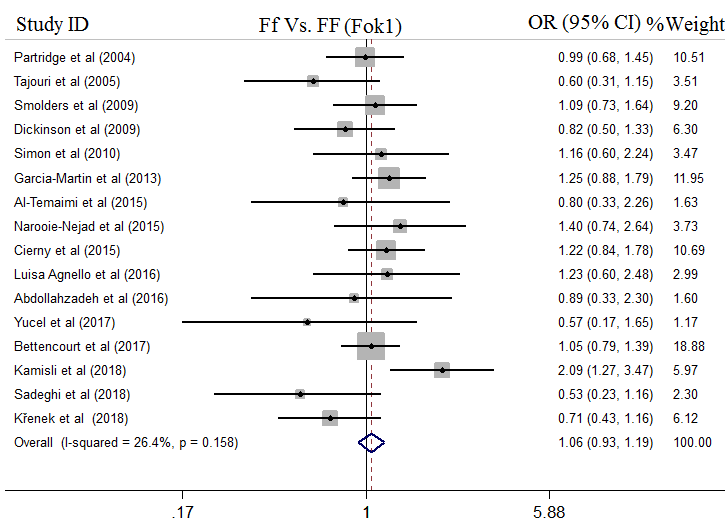


**Supplementary Figure 1.** Forest plot of association between Fok1 gene Polymorphism and MS risk; Dominant model, Recessive model allelic model, ff VS. FF model, Ff vs FF model.


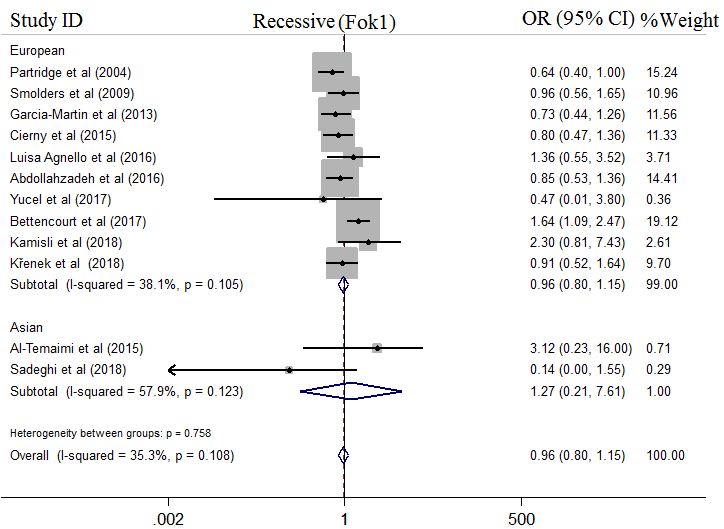

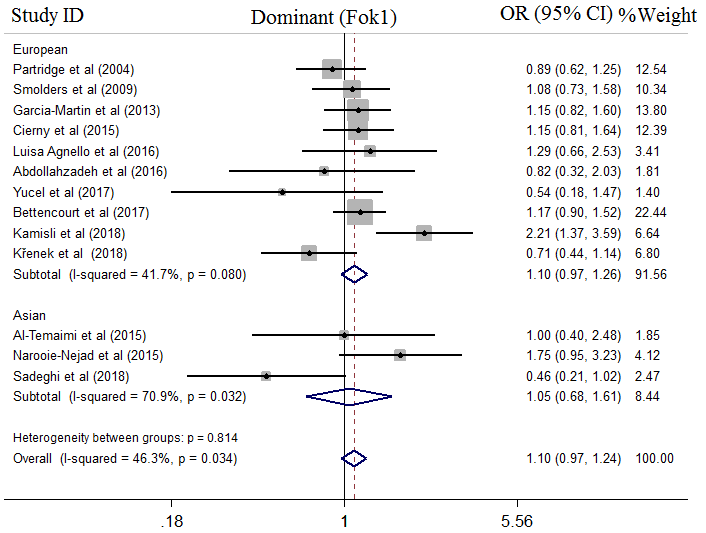


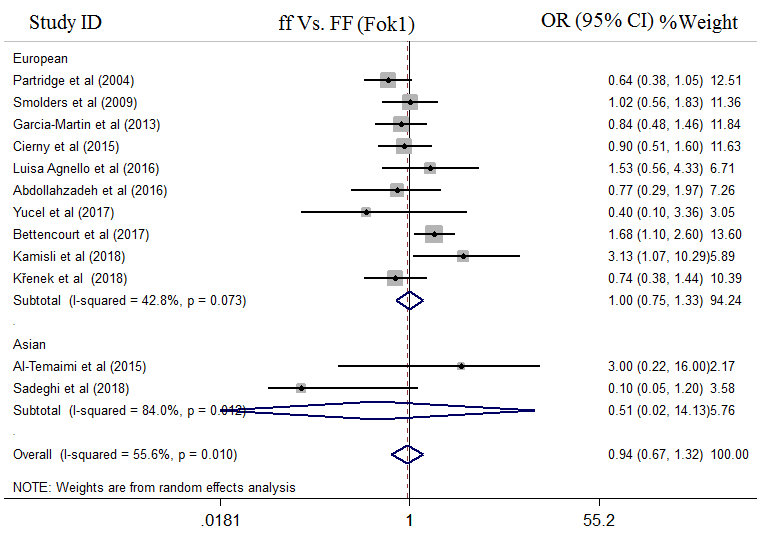

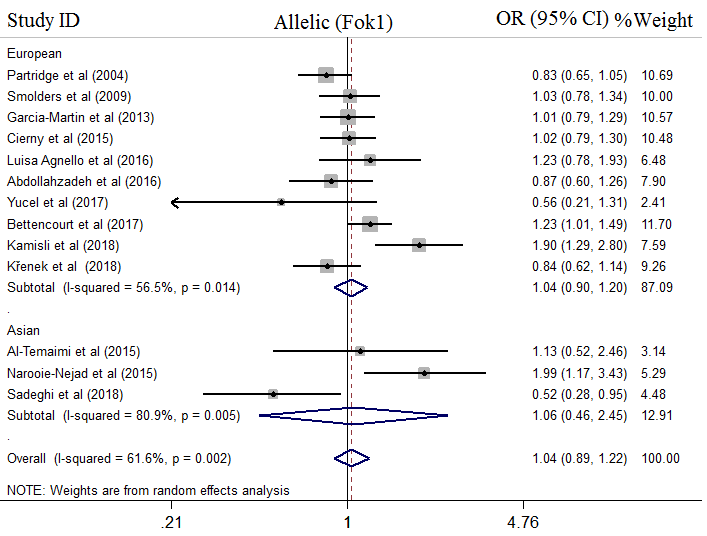


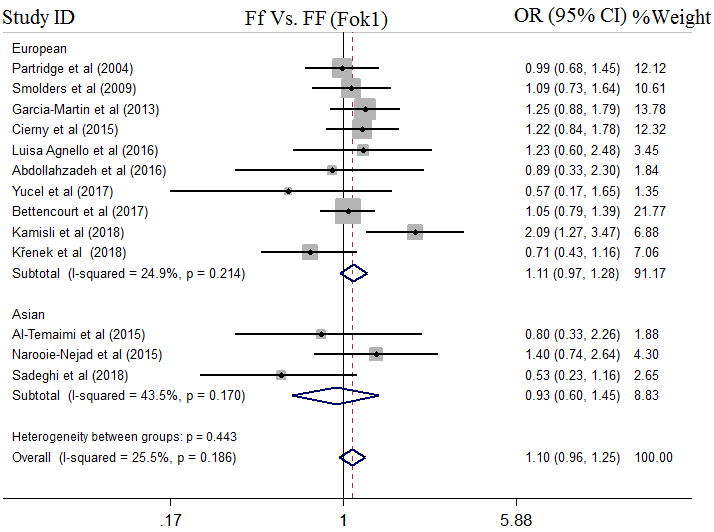


**Supplementary Figure 2.** Forest plot of pooled odds ratio (OR) ) and 95% confidence interval of individual studies and pooled data for the association between Fok1 polymorphism and MS risk in different ethnicity subgroups and overall populations for Dominant model, Recessive model, allelic model, ff VS. FF model, Ff vs FF model.


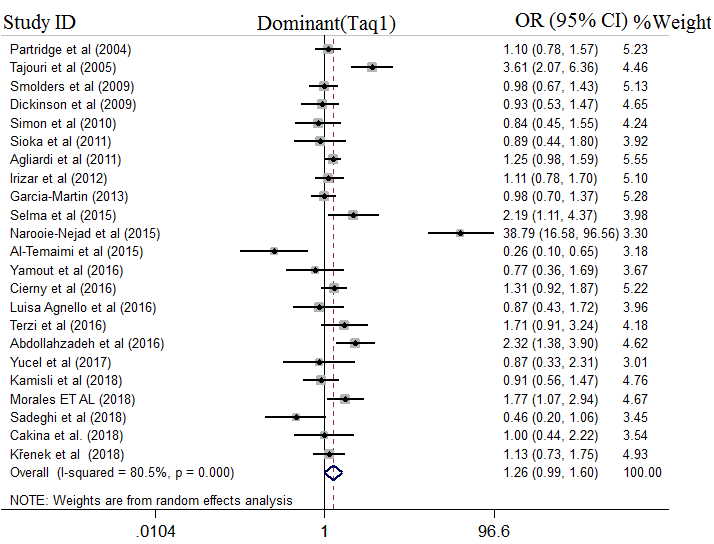

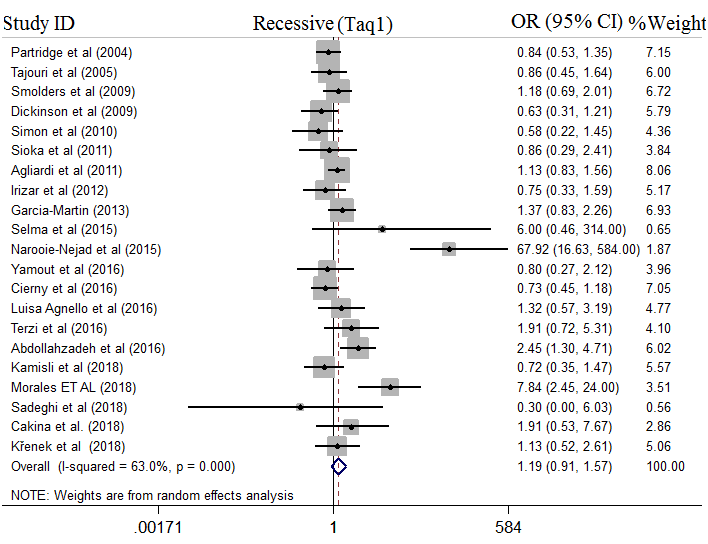


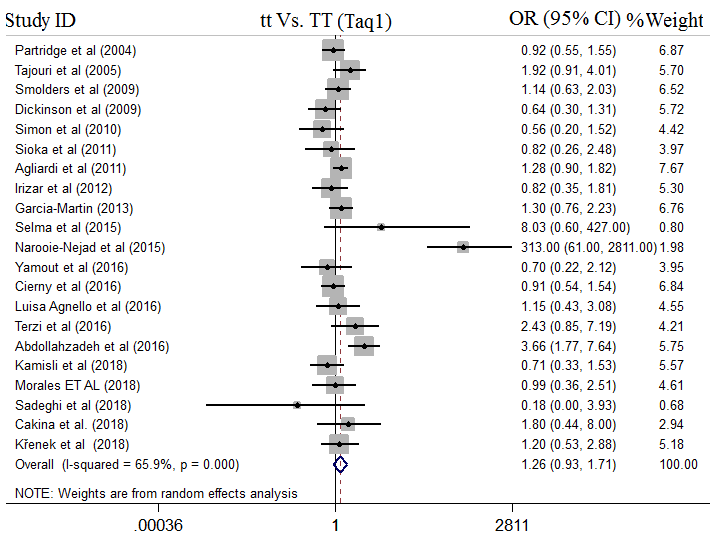

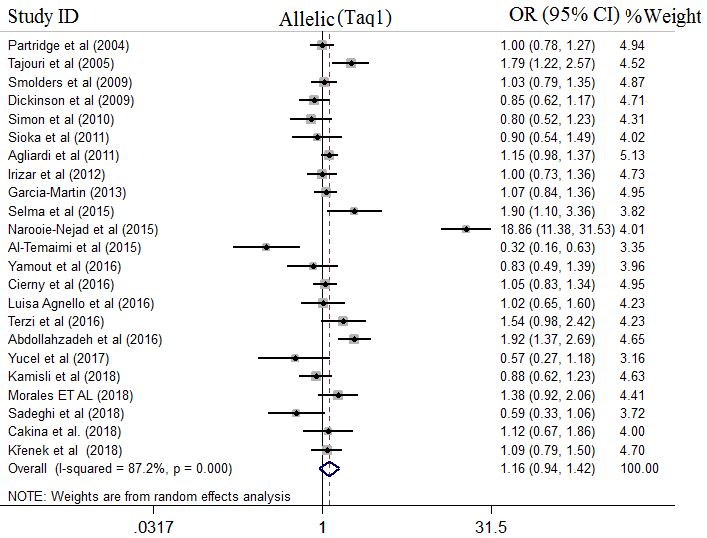


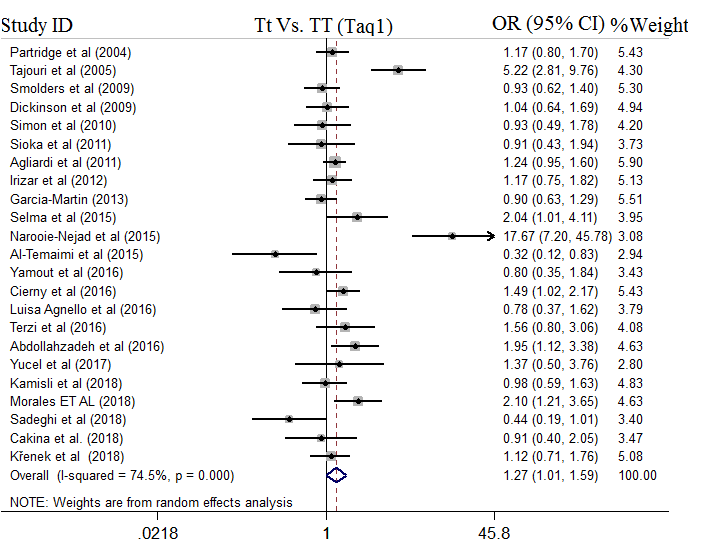


**Supplementary Figure 3.** Forest plot of association between Taq1 gene Polymorphism and MS risk; Dominant model, Recessive model, allelic model, tt VS. TT model, Tt vs TT model.


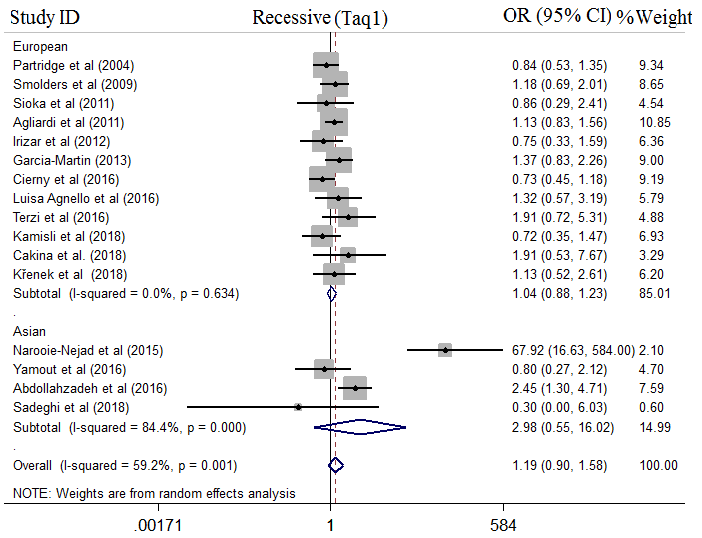

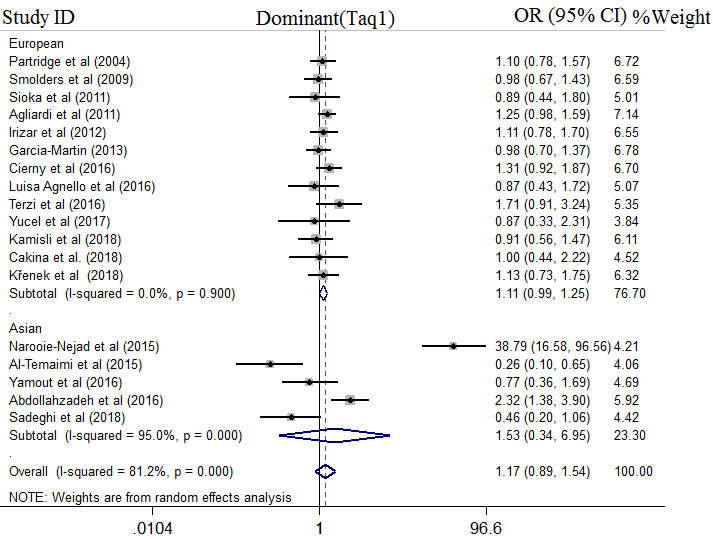


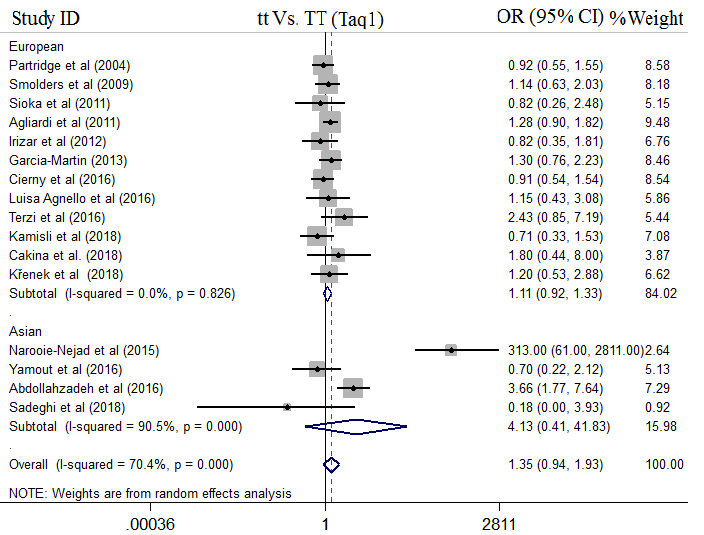

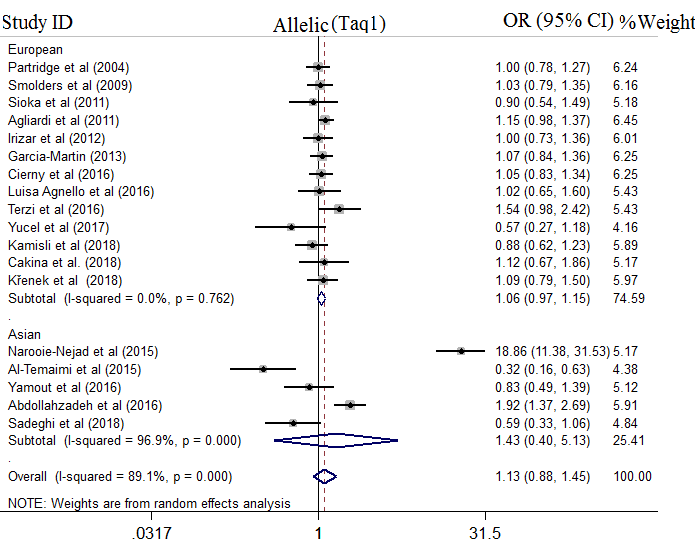


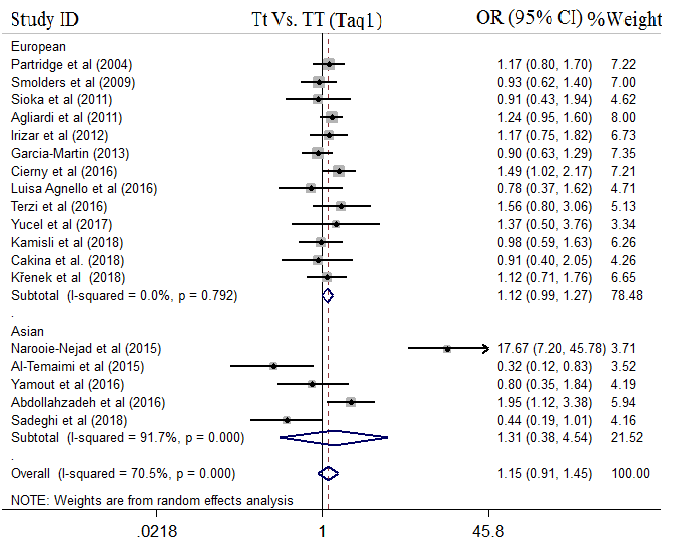


**Supplementary Figure 4.** Forest plot of pooled odds ratio (OR) ) and 95% confidence interval of individual studies and pooled data for the association between Taq1 polymorphism and MS risk in different ethnicity subgroups and overall populations for Dominant model, Recessive model, allelic model, tt vs TT model, Tt vs TT model.


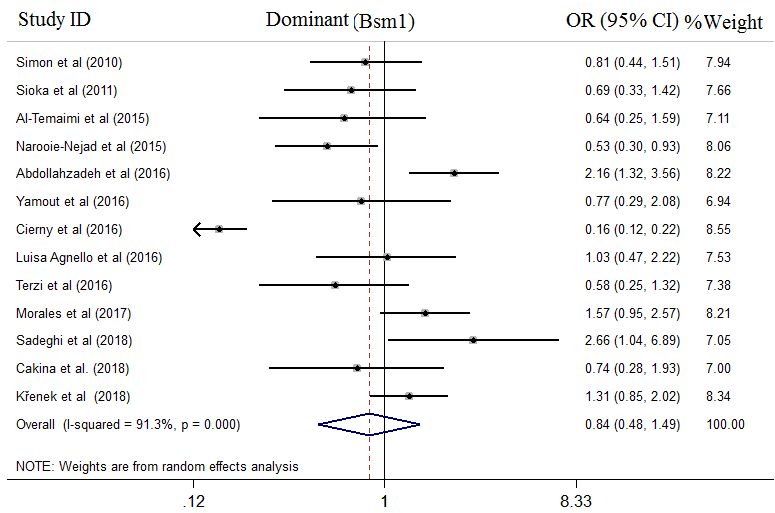

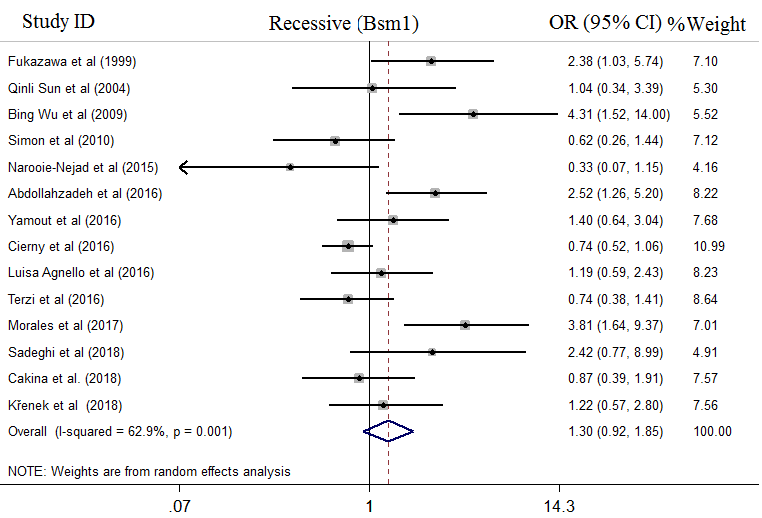


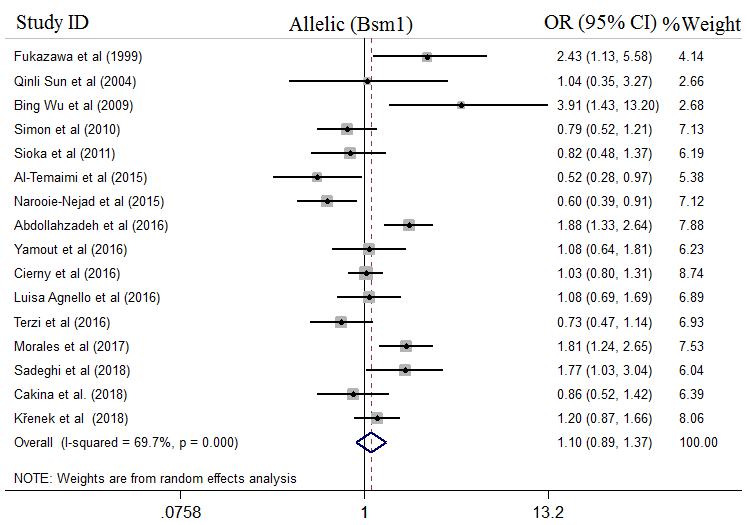

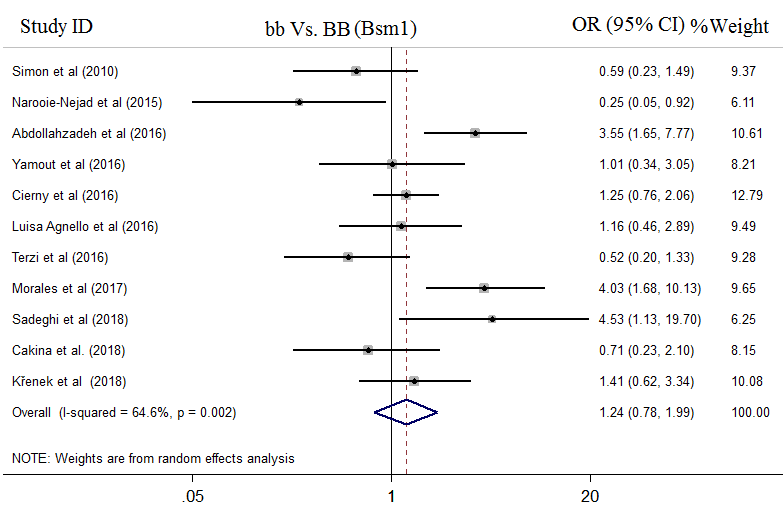


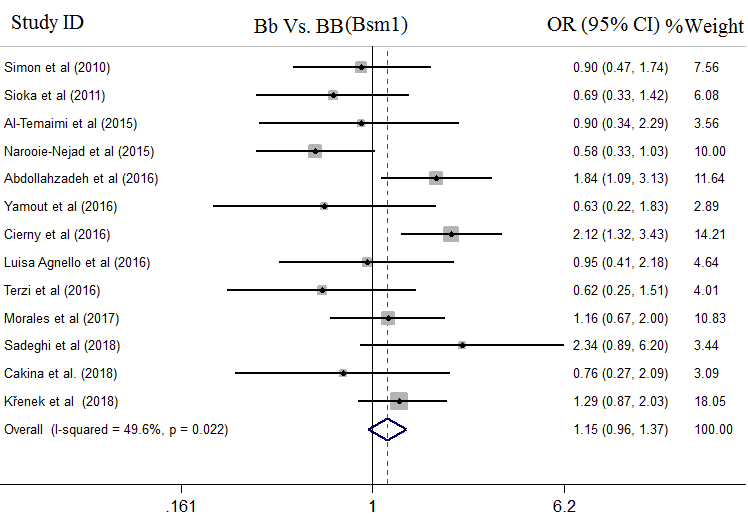


**Supplementary Figure 5.** Forest plot of association between Bsm1 gene Polymorphism and MS risk; Dominant model, Recessive model, allelic model, bb VS. BB model, Bb vs BB model.


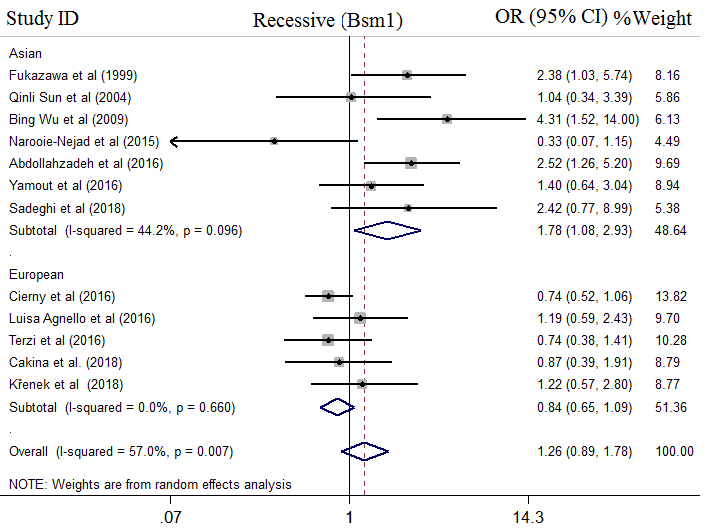

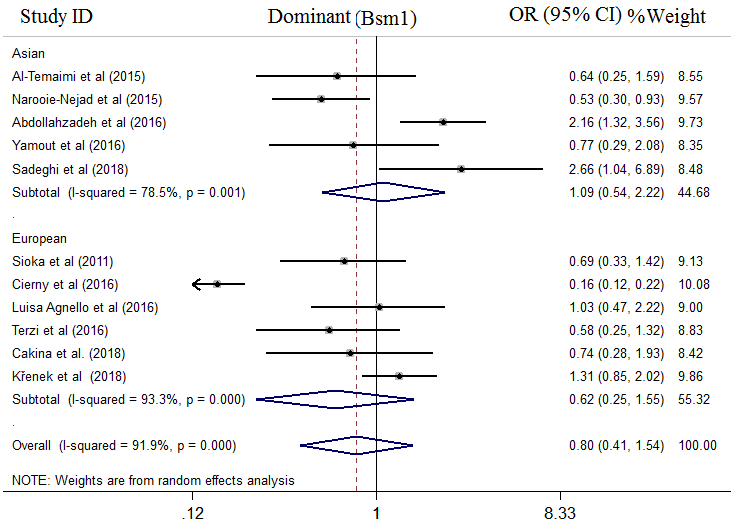


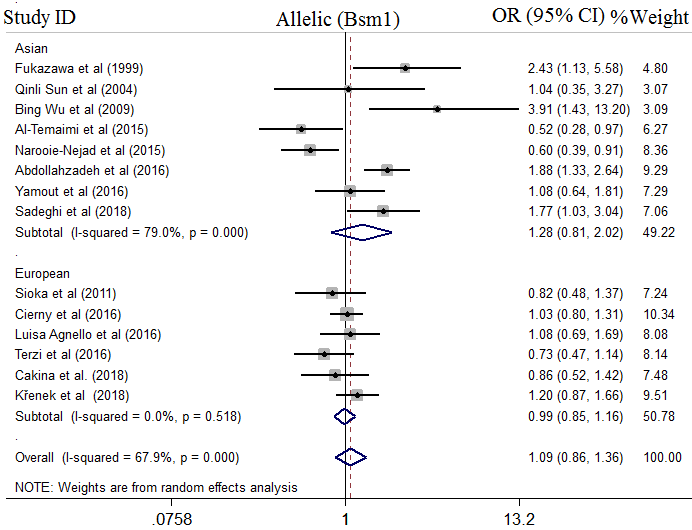

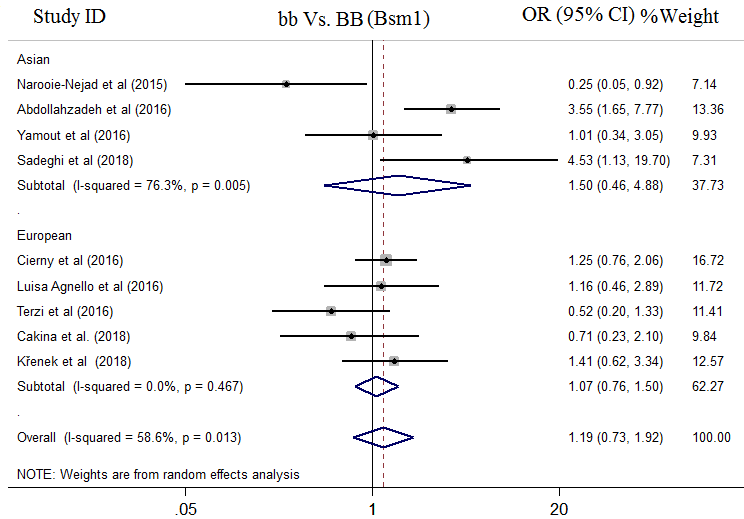


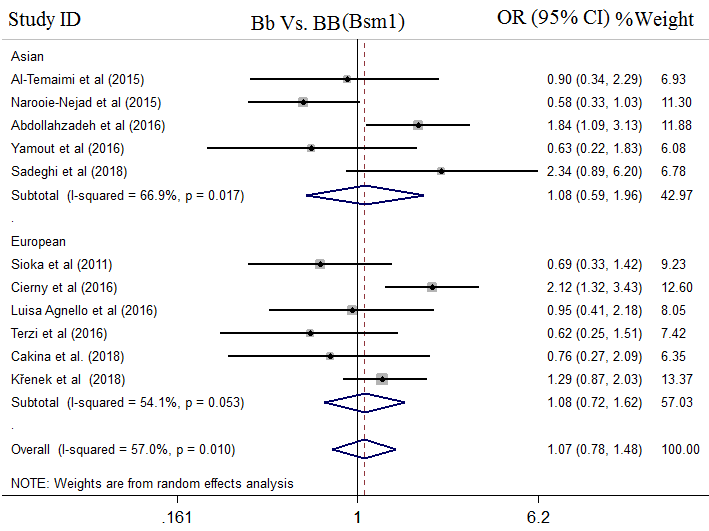


**Supplementary Figure 6 .** Forest plot of pooled odds ratio (OR) ) and 95% confidence interval of individual studies and pooled data for the association between Bsm1polymorphism and MS risk in different ethnicity subgroups and overall populations for Dominant model, Recessive model allelic model, bb VS. BB model, Bb vs BB model.


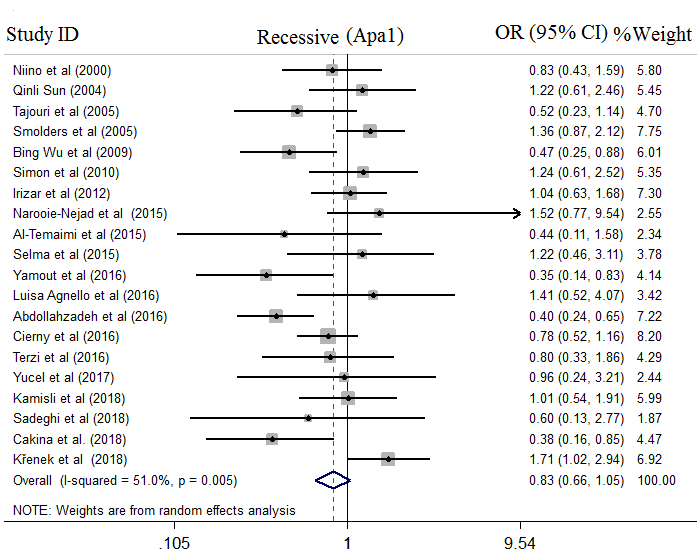

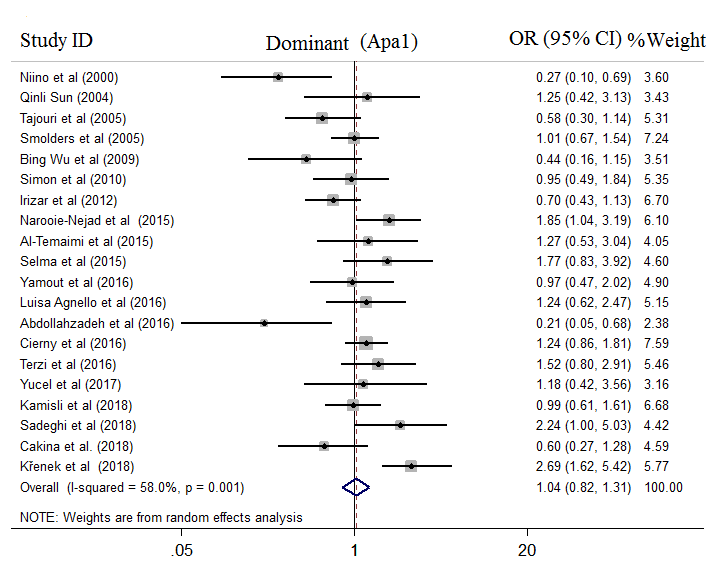


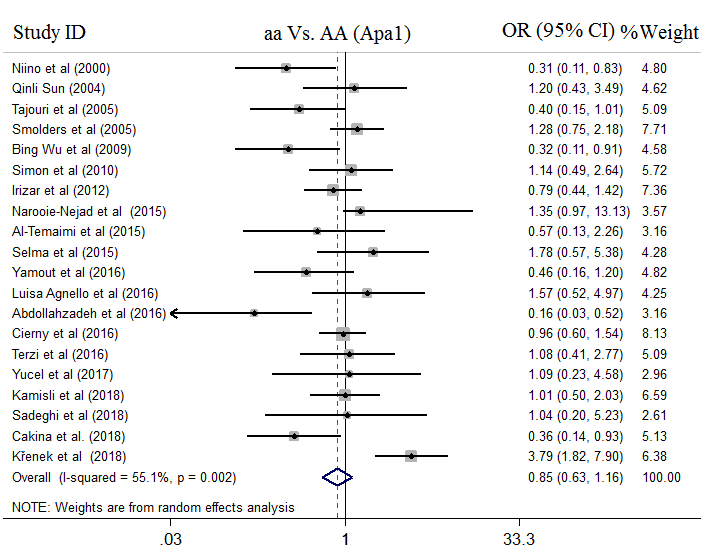

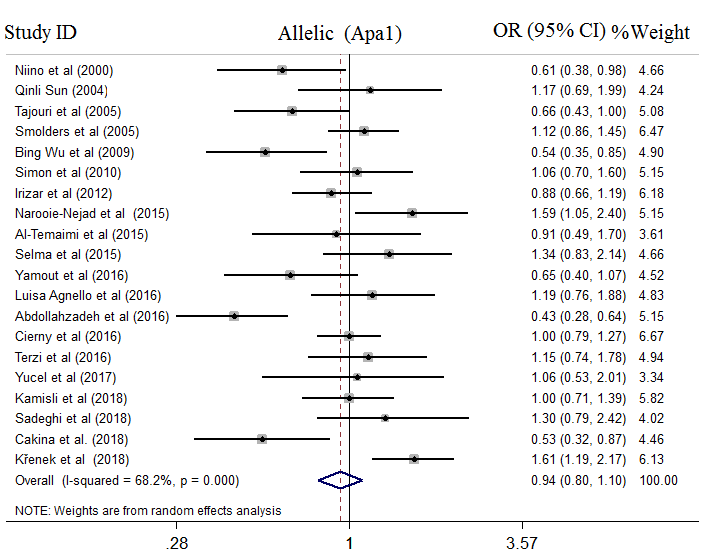


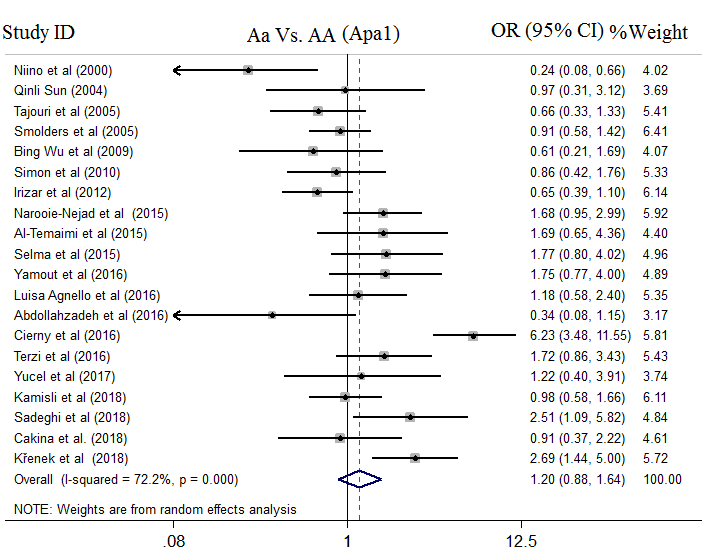


**Supplementary Figure 7.** Forest plot of association between Apa1 gene Polymorphism and MS risk; Dominant model, Recessive model, allelic model, aa VS. AA model, Aa vs AA model.


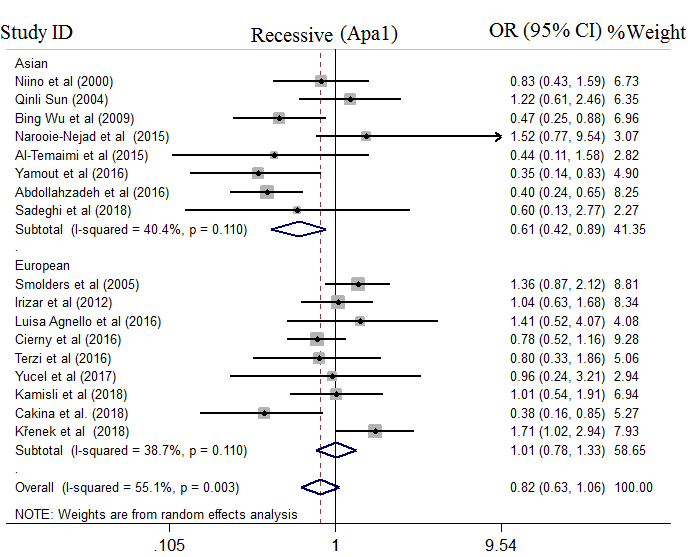

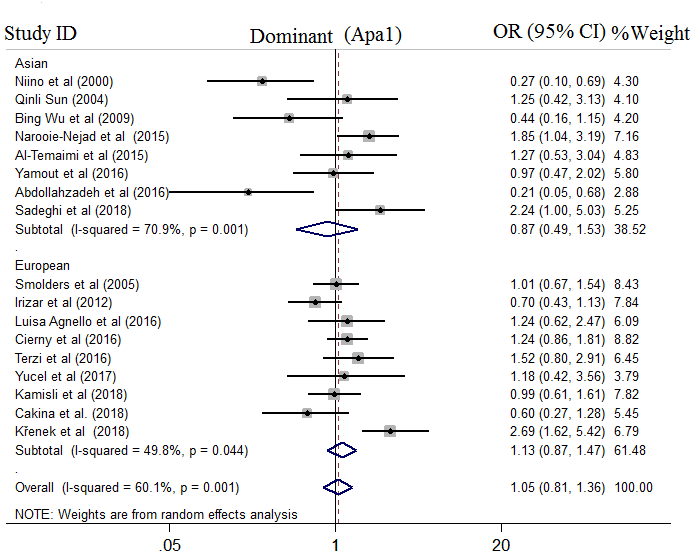


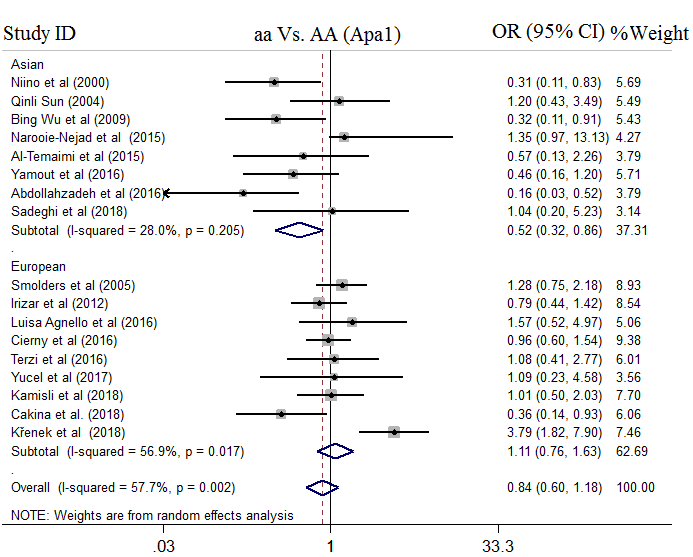

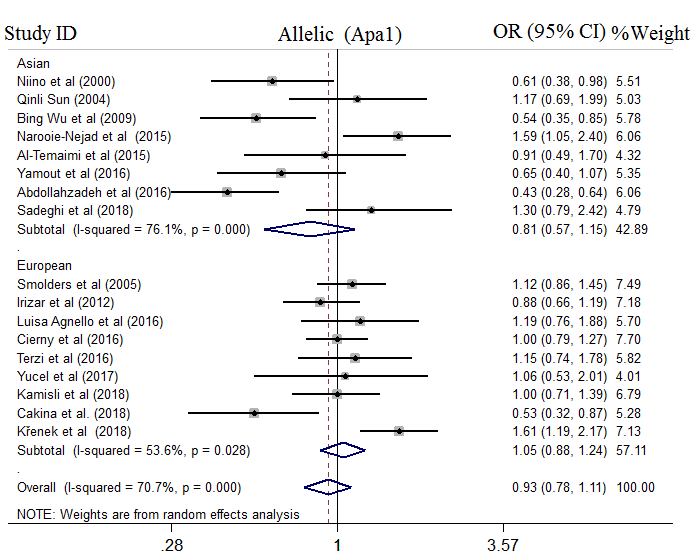


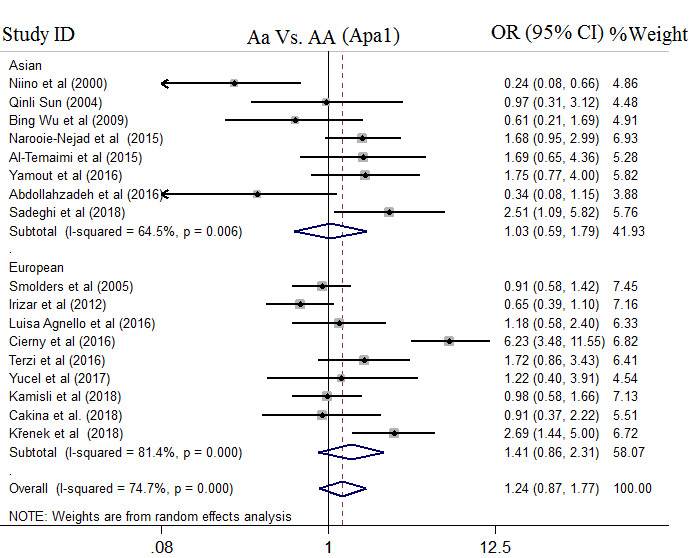


**Supplementary Figure 8.** Forest plot of pooled odds ratio (OR) ) and 95% confidence interval of individual studies and pooled data for the association between Apa1 polymorphism and MS risk in different ethnicity subgroups and overall populations for Dominant model, Recessive model, allelic model, aa VS. AA model, Aa vs AA model.
